# Programming *Bordetella pertussis* lipid A to promote adjuvanticity

**DOI:** 10.1186/s12934-024-02518-7

**Published:** 2024-09-14

**Authors:** Yasmine Fathy Mohamed, Rachel C. Fernandez

**Affiliations:** 1 Department of Microbiology & Immunology, The University of British Columbia, Vancouver, British Columbia V6T1Z3 Canada; 2 Department of Microbiology & Immunology, Faculty of Pharmacy, Alexandria University, Alexandria, 21521 Egypt

**Keywords:** Lipid A modification, Adjuvant, Vaccine, MPLA, *Bordetella pertussis*, Lipopolysaccharide

## Abstract

**Background:**

*Bordetella pertussis* is the causative agent of whooping cough or pertussis. Although both acellular (aP) and whole-cell pertussis (wP) vaccines protect against disease, the wP vaccine, which is highly reactogenic, is better at preventing colonization and transmission. Reactogenicity is mainly attributed to the lipid A moiety of *B. pertussis* lipooligosaccharide (LOS). Within LOS, lipid A acts as a hydrophobic anchor, engaging with TLR4-MD2 on host immune cells to initiate both MyD88-dependent and TRIF-dependent pathways, thereby influencing adaptive immune responses. Lipid A variants, such as monophosphoryl lipid A (MPLA) can also act as adjuvants. Adjuvants may overcome the shortcomings of aP vaccines.

**Results:**

This work used lipid A modifying enzymes from other bacteria to produce an MPLA-like adjuvant strain in *B. pertussis*. We created *B. pertussis* strains with distinct lipid A modifications, which were validated using MALDI-TOF. We engineered a hexa-acylated monophosphorylated lipid A that markedly decreased human TLR4 activation and activated the TRIF pathway. The modified lipooligosaccharide (LOS) promoted IRF3 phosphorylation and type I interferon production, similar to MPLA responses. We generated three other variants with increased adjuvanticity properties and reduced endotoxicity. Pyrogenicity studies using the Monocyte Activation Test (MAT) revealed that these four lipid A variants significantly decreased the IL-6, a marker for fever, response in peripheral blood mononuclear cells (PBMCs).

**Conclusion:**

These findings pave the way for developing wP vaccines that are possibly less reactogenic and designing adaptable adjuvants for current vaccine formulations, advancing more effective immunization strategies against pertussis.

**Supplementary Information:**

The online version contains supplementary material available at 10.1186/s12934-024-02518-7.

## Introduction

Pertussis is a highly contagious severe acute respiratory illness that is easily transmitted in humans through airborne respiratory droplets [1, 2]. Pertussis resurgence represents a serious global health concern [3, 4]. Annually, an estimated 24 million cases of pertussis occur worldwide, with over 160,000 deaths occurring in children despite the high vaccination coverage [5].

The current approach to combating pertussis involves two types of vaccines: whole-cell pertussis (wP) and acellular pertussis (aP). While the wP vaccine, introduced in the late 1940s, significantly reduced pertussis rates, its high reactogenicity led to decreased acceptance [6]. Over two decades ago, concerns about the wP vaccine’s safety prompted its replacement with aP vaccines, which have fewer side effects [7, 8]. While the aP vaccines provide protection against pertussis in most cases, recent cases of pertussis in fully vaccinated children are linked to aP vaccine’s failure to prevent colonization and provide long-term immunity [9]. This may be due vaccine selection pressure leading to antigenic alteration in the circulating *B. pertussis* strains, waning immunity, and limited antigenic components (1–5) in aP vaccines [10]. Studies suggest that wP vaccines are more effective in inducing protective responses than aP vaccines [11–13] highlighting the need for new-generation pertussis vaccine candidates.

Lipopolysaccharide (LPS) serves as the primary constituent of the outer leaflet of Gram-negative bacteria, comprising discrete structural regions including lipid A, core oligosaccharide (core), and in many bacteria, the repeating O-antigen units [14]. Lipid A is the bioactive anchor of LPS and functions as a potent ligand for Toll-like receptor 4 (TLR4)/myeloid differentiation factor 2 (MD-2) receptor of the innate immune system stimulating the host immune responses [15, 16]. This activation can then signal via two distinct pathways, the MyD88-dependent and the Toll/IL-1 receptor domain-containing adapter inducing interferon-β (TRIF)-dependent pathways, thereby influencing adaptive immune responses [17]. In the MyD88-dependent pathway, dimerization of TLR4/MD-2 activates the transcription factor NFκB triggering the release of pro-inflammatory cytokines [17]. Additionally, TLR4 can be internalized into endosomes activating the TRIF pathway, which ultimately results in the activation of the IRF3 transcription factor, the production of type I interferons and activation of dendritic cells, thereby, playing an important role in the stimulation of early T-cell responses [18, 19]. Type I interferons such as IFN-$\:\beta\:$ exhibit adjuvant-like properties [20]. Hence, LPS can act as an endotoxin as well as an adjuvant based on the type of cytokines produced.

**Fig. 1:**
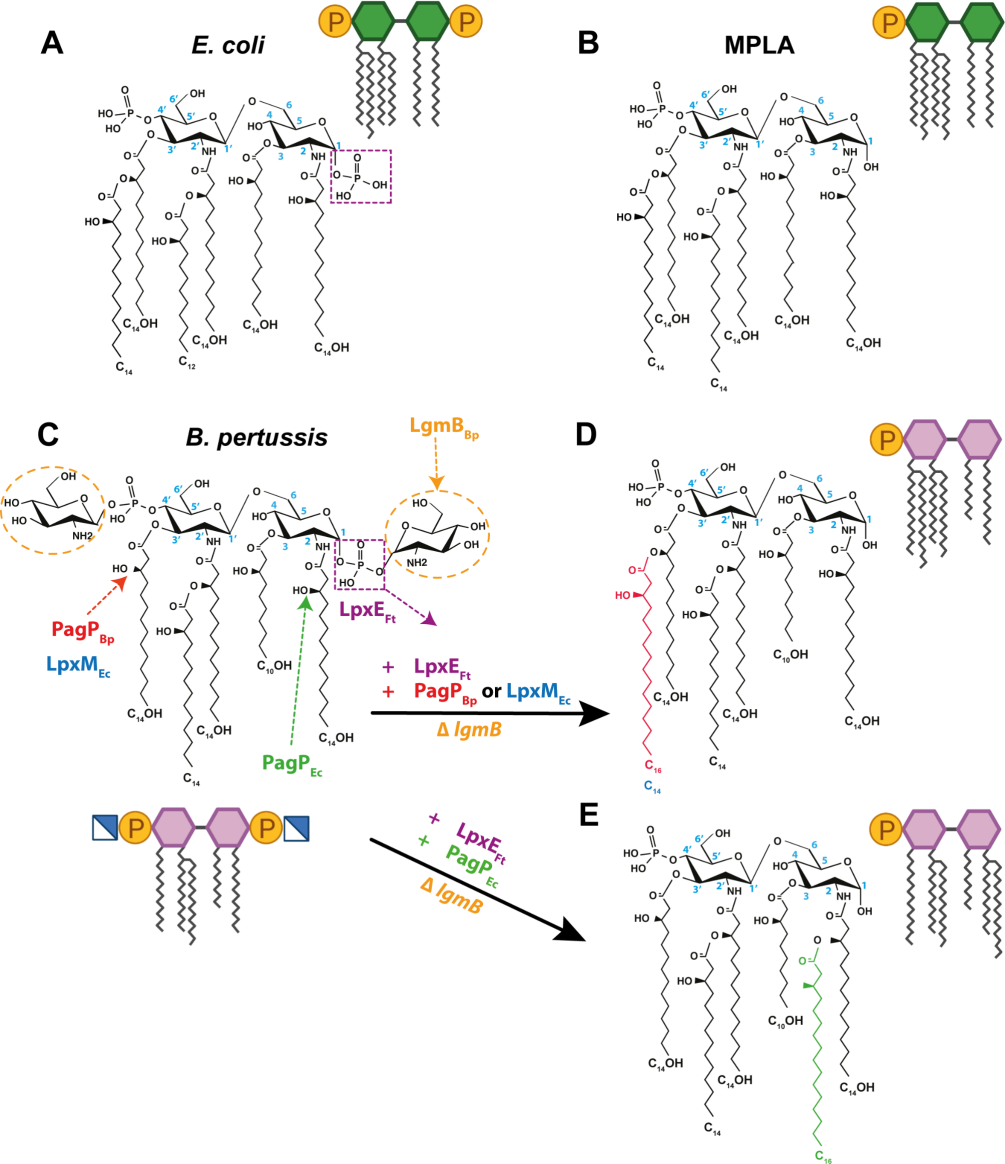
Chemical structure of lipid A. Hexa-acylated lipid A species from *E. coli* (**A**), synthetic monophosphoryl lipid A; MPLA (**B**), penta-acylated glucosamine-modified lipid A from *B. pertussis* (strain BP338, a Tohama I derivative) (**C**). Conversion of *B. pertussis* lipid A into MPLA-like structure requires deletion of *lgmB* and introduction of the 2 enzymes, LpxE and PagP or LpxM (**D** and **E**). The lgmB mutant lacks the GlcN residues (dotted circles), LpxE removes the phosphate group from the C1-position (dotted rectangle), PagP_Bp_ and LpxM_Ec_ add a secondary acyl chain (palmitate and myristate; respectively) to the C3’ position. PagP_Ec_ adds a secondary palmitate to the C2 position. Proposed structures are based on enzyme function. Structure diagram was modified from [23]

The lipooligosaccharide (LOS) of *B. pertussis*, lacking the typical O antigen and featuring a short trisaccharide, is composed of lipid A and a branched oligosaccharide core [21, 22]. Its lipid A is composed of a penta-acylated di-glucosamine backbone, in which the phosphate groups are substituted with glucosamine residues (GlcN) leading to heightened proinflammatory responses [23]. Alterations in acyl chains or phosphate groups of lipid A can impact TLR4 activation, directing the signaling pathway towards either the highly proinflammatory MyD88 dependent pathway or the less inflammatory TRIF pathway [18, 24]. For instance, *Escherichia coli* lipid A, with six acyl chains and two phosphate groups is a potent TLR4 agonist with strong endotoxic activity (Fig. 1A). Conversely, monophosphoryl lipid A (MPLA), lacking a single phosphate at C1 position (Fig. 1B) serves as an effective adjuvant promoting desirable Th1-biased immune responses in various approved vaccines [18, 25, 26].

This concept of modifying the structural composition of lipid A can be harnessed for the development of novel vaccine candidates [24] and adjuvants, including those for pertussis. In this study, we manipulated *B. pertussis* by modifying its lipid A structure, to generate potential novel wP vaccine candidates and adjuvants. We explored the feasibility of developing an intrinsic adjuvant strain with MPLA-like properties (Fig. 1) that could offer enduring immunity [27] and be less reactogenic, without relying on MPLA itself as an adjuvant. Along these lines, LOS from these strains could also serve as adjuvants for aP vaccines. Our approach involved utilizing combinations of heterologous bacterial enzymes [24] to modify the lipid A of *B. pertussis* in the number, position, and length of acyl chains, as well as its phosphorylation state, and subsequently assessing the distinct TLR4 responses resulting from these modifications. We introduced a range of lipid A modifications in both the *B. pertussis* BP338 strain and its GlcN mutant strain ($\:\varDelta\:$*lgmA-D*). We generated a hexa-acylated monophosphorylated lipid A (Fig. 1E) that does not possess the exact structure of MPLA (Fig. 1D) yet exhibits comparable responses resembling those of MPLA. We also discovered three additional distinct lipid A structures, which demonstrated adjuvant properties despite being different from MPLA. Our findings highlight the engineering of four lipid A variants that possess reduced endotoxicity and increased adjuvanticity that can be further developed as adjuvants for the next generation of pertussis vaccines. Although bacterial strains themselves with these lipid A modifications exhibited higher NFκB activation, they induced a TRIF-biased response and could be further investigated for their potential as novel wP vaccines, which are still used in low and middle-income countries. By focusing on manipulating LOS (lipid A) we address improvements to both whole-cell and acellular pertussis vaccines.

## Results

### Generation of different LOS variants in *B. pertussis* and identification of select incompatibilities of lipid A modifying enzymes

The structure of lipid A can vary amongst Gram negative bacteria. This is controlled by different enzymes that modify structural features such as the length, number of acyl chains and number of phosphate groups on lipid A [24]. In this study we generated diverse lipid A variants by introducing lipid A modifying enzymes from different bacteria into *B. pertussis*, that has or lacks the glucosamine (GlcN) modification [28]. We developed distinct lipid A variants in the wild-type *B. pertussis* strain BP338 as well as the GlcN mutant to study the combined effect of lipid A modifying enzymes together with the absence of glucosamine modification on endotoxicity. Foreign genes were delivered into *B. pertussis* by a low-copy plasmid that encodes a *B. pertussis* heat shock promoter [28] or a tet-inducible promoter (pIG10) developed in our lab (Ifill and Fernandez, manuscript in preparation). Genes of interest were cloned separately or in tandem into selected plasmids and introduced into *B. pertussis* by diparental mating. Plasmid-encoded genes were maintained by selection antibiotics.

MPLA is a U.S. Food and Drug Administration (FDA) approved human adjuvant in licensed vaccines [29] with valuable immunostimulatory properties [26]. The structure of MPLA, depicted in Fig. 1B, differs from the lipid A of *E. coli* (Fig. 1A) as it lacks the phosphate group at C1. Our primary objective was to create a built-in adjuvant strain resembling MPLA by deleting *lgmB* and incorporating the following enzymes: PagP_Bp_ or LpxM_Ec_, leading to the production of a hexa-acylated lipid A species, and LpxE_Ft_, which generates a monophosphorylated lipid A (Fig. 1C and D).

**Fig. 2:**
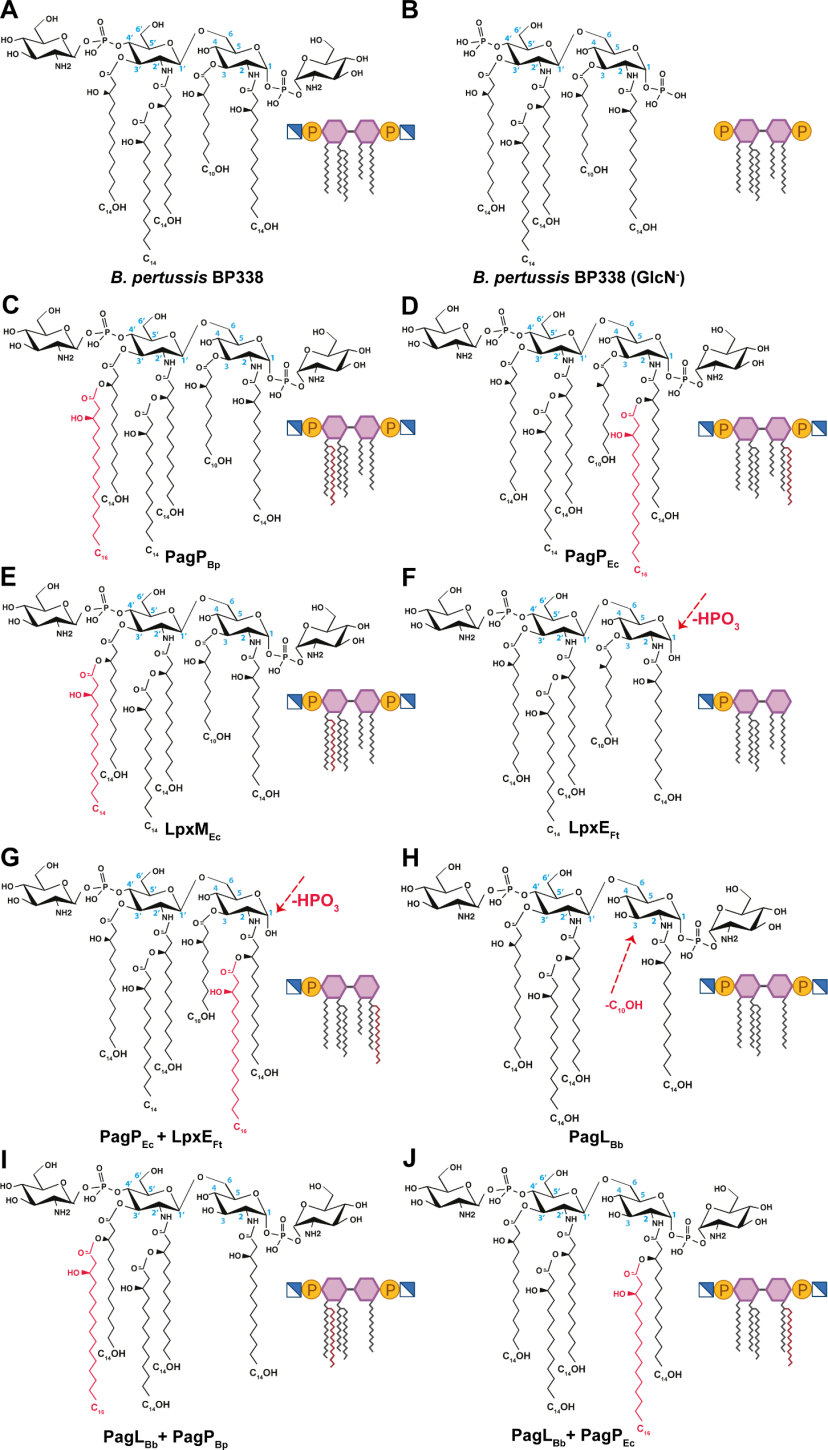
Expected lipid A structures after introducing lipid A modifying enzymes. Lipid A structures of *B. pertussis* wild-type strain BP338 (**A**), its isogenic GlcN mutant $\:\varDelta\:$*lgmA-D* (**B**), PagP_Bp_ (**C**), PagP_Ec_ (**D**), LpxM_Ec_ (**E**), LpxE_Ft_ (**F**), PagP_Ec_ + LpxE_Ft_ (**G**), PagL_Bb_ (**H**), PagL_Bb_ + PagP_Bp_ (**I**) and PagL_Bp_ + PagP_Ec_ (**J**). The modifications in the lipid A structure are shown in red. The heterologous enzymes expressed are indicated below the structures. Bp: *B. pertussis*, Ec: *E. coli*, Ft: *Francisella tularensis*, and Bb: *B. bronchiseptica*

Figure 2 illustrates the lipid A variants created in *B. pertussis* based on enzyme function. LgmB, an inner membrane enzyme, adds GlcN substituents to phosphate groups (Fig. 2A and B) [23]. In our experimental approach, we employed PagP_Bp_ from *B. pertussis* to introduce a palmitate secondary acyl chain at C3’ (Fig. 2C), and PagP_Ec_ from *E. coli* to add a palmitate at C2 (Fig. 2D) [30, 31]. Expression of PagP_Bp_ in *B. pertussis* is abolished by the presence of an insertion sequence element in the promoter region [32]. So, in our work the *pagP*_*Bp*_ gene was cloned without its promoter and its expression was regulated by the heat shock promoter in the pBBR2pcpn plasmid. LpxM_Ec_, which catalyzes the myristoylation of lipid A at the 3’ position, was also included (Fig. 2E) [33]. We also incorporated LpxE which is an inner membrane phosphatase that dephosphorylates lipid A on the periplasmic surface of the inner membrane and has been reported in *Francisella tularensis* and *F. novicida*. LpxE_Ft_ was used to eliminate the phosphate at the C1 position (Fig. 2F) [34].

We encountered compatibility issues when co-expressing PagP_Bp_ or LpxM_Ec_ with LpxE_Ft_ in *B. pertussis* to achieve hexa-acylated, monophosphorylated lipid A.  We attempted various strategies, including expressing both enzymes on the same or separate plasmids, experimenting with different plasmid combinations, and using chemical treatments to enhance substrate accessibility [35]. Despite our efforts, we couldn’t achieve the desired construct. Consequently, we opted for using PagP_Ec_ in combination with LpxE_Ft_ to generate an MPLA-like structure with a unique acyl chain arrangement (Fig. 1E). To introduce the desired modifications, we expressed PagP_Ec_ and LpxE_Ft_ either on separate plasmids (PagP_Ec_ + LpxE_Ft_) or on the same plasmid (PagP_Ec_ / LpxE_Ft_) using FastCloning techniques (Fig. 2G). Furthermore, PagL_Bb_ from *B bronchiseptica*, which hydrolyzes the ester bond at the C3 position of lipid A, releasing the acyl chain at this location, was included in our modifications (Fig. 2H) [36]. The open reading frame of PagL_Bp_ in *B. pertussis* is disrupted by a frameshift, so, we used PagL_Bb_ from *B. bronchiseptica* instead which has an intact open reading frame [31, 37]. Both PagL and PagP are outer membrane enzymes [38]. Additionally, we generated other penta-acylated lipid A variants with different acyl chain positions by co-expressing PagL_Bb_ with either PagP_Bp_ or PagP_Ec_, resulting in PagL_Bb_ + PagP_Bp_ and PagL_Bb_ + PagP_Ec_, respectively (Fig. 2I and J). All lipid A modifications illustrated in Fig. 2 were made in both *B. pertussis* BP338 and GlcN mutant strains.

### MALDI-TOF analysis of recombinant lipid A structures

Lipid A was extracted by the isobutyric acid-ammonium hydroxide hydrolysis method from whole bacterial cells [39]. The structures of the engineered lipid A constructs were confirmed through mass spectrometry (MS) analysis utilizing matrix-assisted laser desorption/ionization time of flight (MALDI-TOF).

**Fig. 3:**
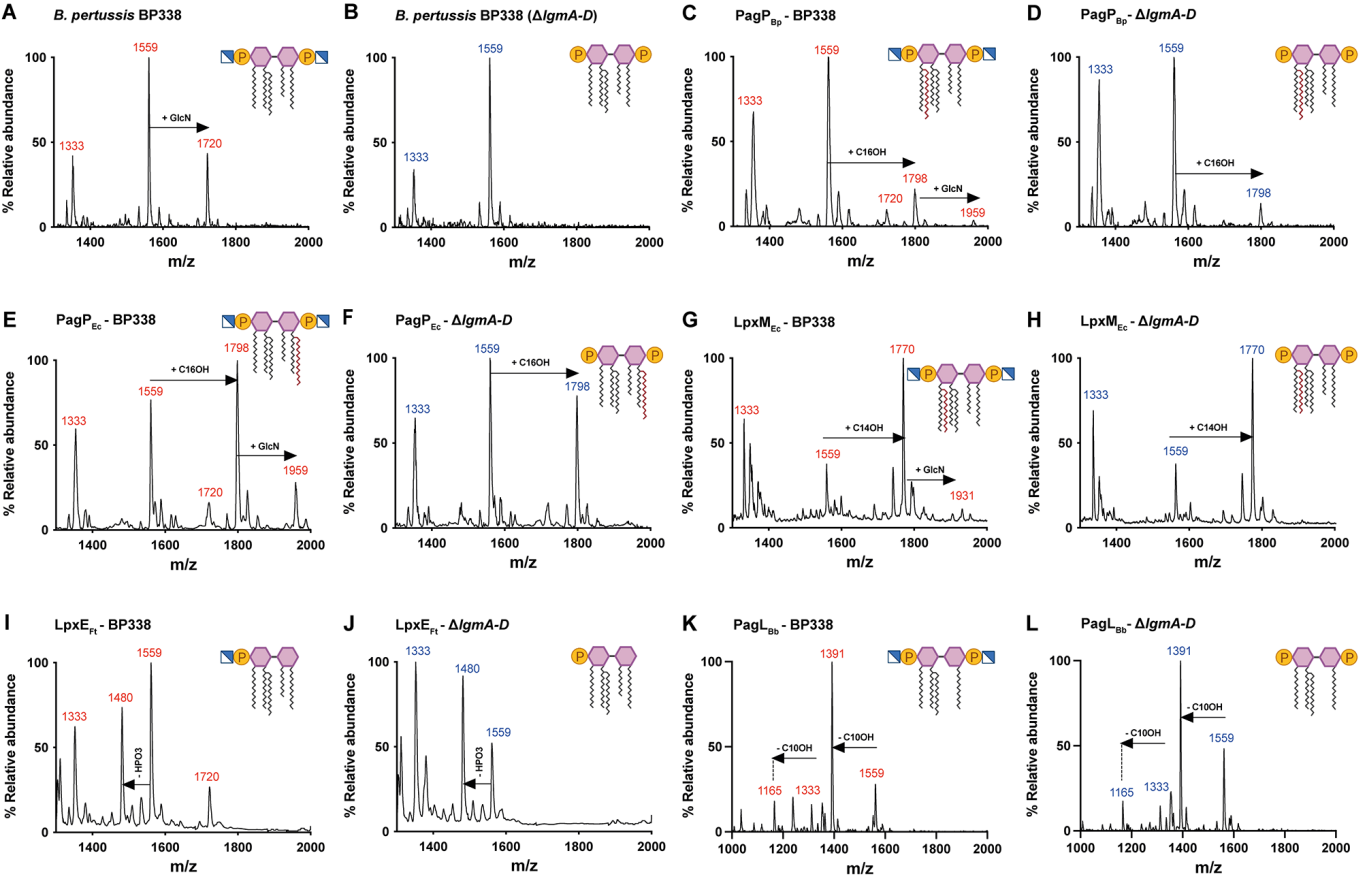
Structural analysis of lipid A of strains expressing single lipid A modifying enzymes by MALDI-TOF. Negative-ion MALDI-TOF mass spectra of lipid A isolated from *B. pertussis* BP338 (**A**), ∆*lgmA-D* (**B**), PagP_Bp_ (**C** and **D**), PagP_Ec_ (**E** and **F**), LpxM_Ec_ (**G** and **H**), LpxE_Ft_ (**I** and **J**) and PagL_Bb_ (**K** and **L**)

In the wild-type *B. pertussis* strain BP338, a major peak at m/z 1559 indicates bis-phosphorylated penta-acylated lipid A, while an m/z 1720 peak represents phosphate group substitution with GlcN [23](Fig. 3A). This peak was absent in the GlcN mutant strain ($\:\varDelta\:$*lgmA-D*) (Fig. 3B). Strains expressing PagP_Bp_ or PagP_Ec_ exhibited 2 additional peaks: m/z 1798 (hexa-acylated lipid A) and m/z 1959 (hexa-acylated species with GlcN substitution) (Fig. 3C and E). The ion at m/z 1798 indicated the substitution of the primary acyl chain at either position C3’ or C2 with C16-OH by the enzymes PagP_Bp_ or PagP_Ec_, respectively. The peak at m/z 1959 was absent in $\:\varDelta\:$*lgmA-D* (Fig. 3D and F). The strain expressing LpxM_Ec_ showed an m/z 1770 peak, which corresponds to the addition of a secondary acyl chain (C14-OH) to C3’, along with an m/z 1931 peak representing hexa-acylated lipid A species at m/z 1770 with a GlcN substituent (Fig. 3G and H). MALDI-TOF analysis of lipid A from the strain expressing LpxE_Ft_ revealed a peak at m/z 1480, indicating the removal of one phosphate group (HPO3) from BP338 (Fig. 3I) or $\:\varDelta\:$*lgmA-D* (Fig. 3J). Lipid A analysis of the strain expressing PagL_Bb_ showed a peak at m/z 1391, corresponding to tetra-acylated lipid A due to the removal of C10-OH at position C3 (Fig. 3K and L). Surprisingly, no peak corresponded to tetra-acylated lipid A species with a GlcN substituent.

**Fig. 4:**
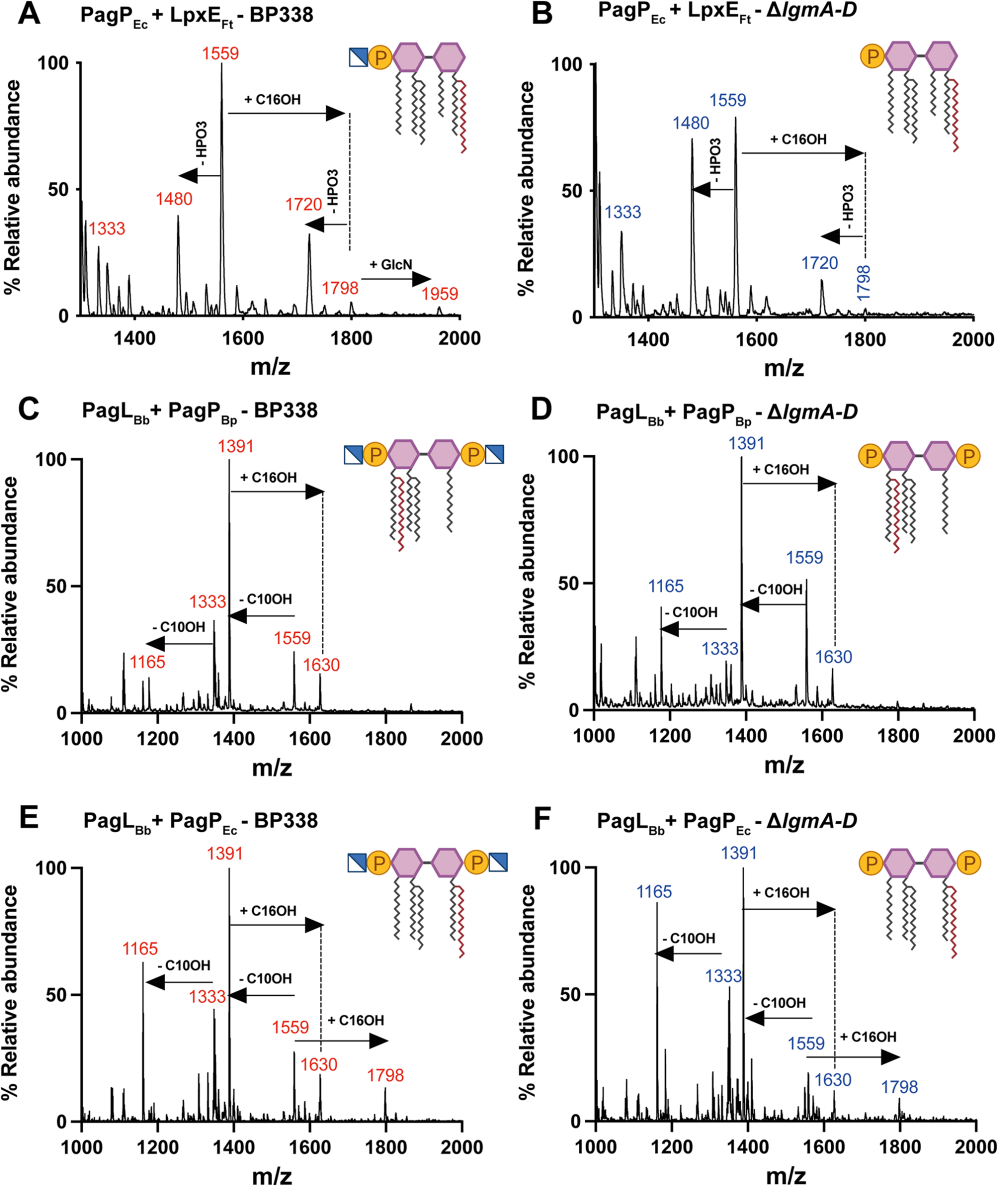
Structural analysis of lipid A of strains expressing two lipid A modifying enzymes by MALDI-TOF. Negative-ion MALDI-TOF mass spectra of lipid A isolated from PagP_Ec_ + LpxE_Ft_ (**A** and **B**), PagL_Bb_ + PagP_Bp_ (**C** and **D**) and PagL_Bb_ + PagP_Ec_ (**E** and **F**)

In the strain expressing both PagP_Ec_ and LpxE_Ft_ on separate plasmids, a prominent peak at m/z 1720 was observed for the hexa-acylated monophosphorylated lipid A species. This peak indicated the loss of a phosphate group from the hexa-acylated species at m/z 1798 (Fig. 4A). Notably, it coincided with the penta-acylated lipid A species with a GlcN substituent. Interestingly, this peak was present in the PagP_Ec_ + LpxE_Ft_ - $\:\varDelta\:$*lgmA-D* strain (Fig. 4B), which lacks the GlcN substituent suggesting it is a hexa-acylated monophosphorylated lipid A species. However, introducing PagP_BP_ or LpxM_Ec_ with LpxE_Ft_ didn’t produce a similar peak (Fig. S1). Despite efforts to induce phospholipid migration with EDTA in the PagP_Bp_ + LpxE_Ft_ strain [35, 38], no peak corresponding to hexa-acylated monophosphorylated lipid A species was detected using MALDI-TOF. Similar peaks were observed from the strain expressing PagP_Ec_ and LpxE_Ft_ cloned in tandem in pIG10 (Supp. Fig. S2).

The expression of both PagL_Bb_ and PagP resulted in the presence of penta-acylated lipid A species represented by a peak at m/z 1630 that corresponded to the addition of C16-OH to the tetra-acylated species at m/z 1391 (Fig. 4C, D, E and F). In the case of the strain expressing PagL_Bb_ and PagP_Ec_, an additional peak at m/z 1798 corresponding to hexa-acylated lipid A species, was observed (Fig. 4E and F).

### TLR4 activation by the LOS variants

**Fig. 5:**
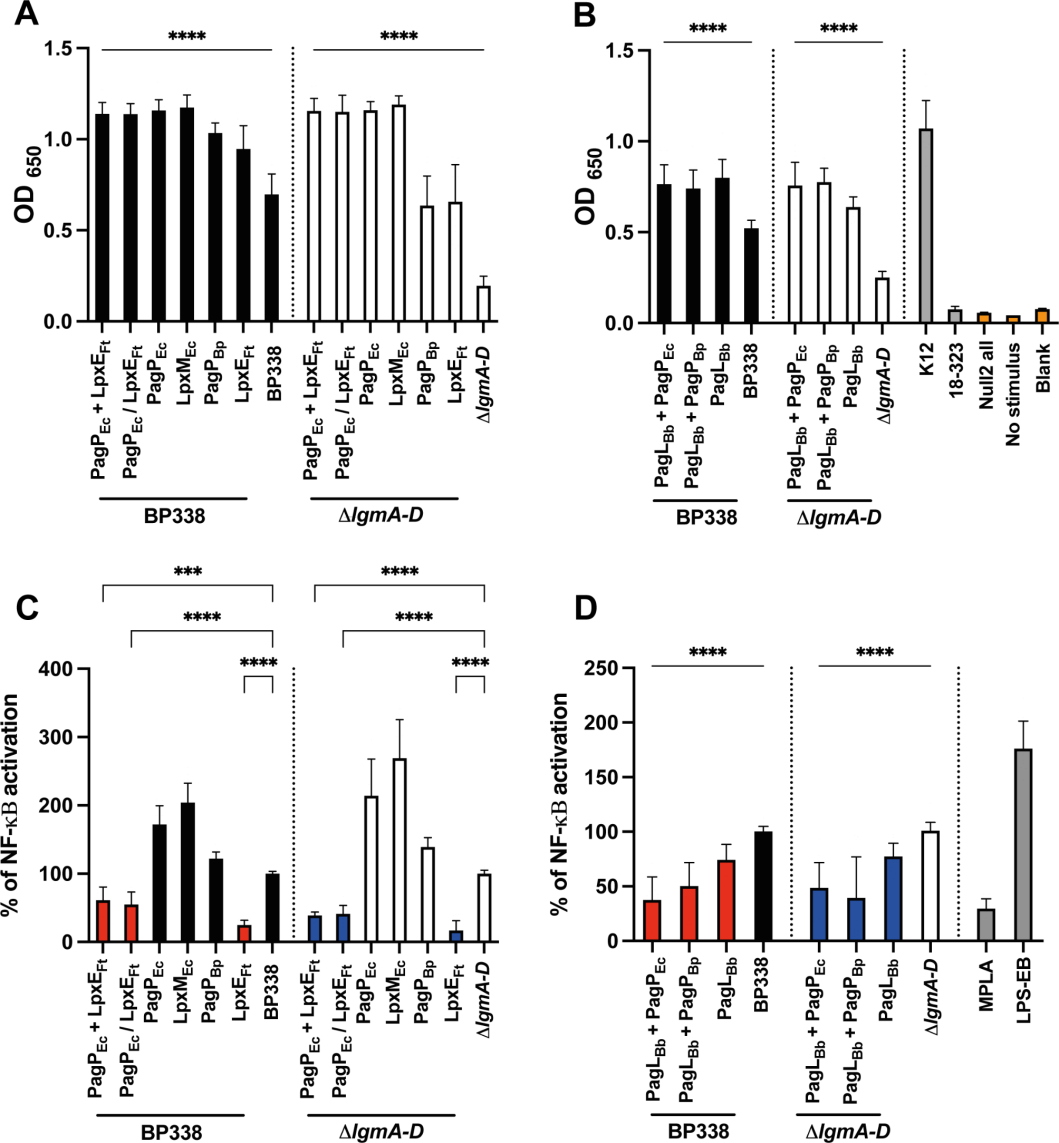
TLR4 activity as measured with the HEK-Blue NFκB TLR4 activity assay. HEK-Blue hTLR4 cells were stimulated with *B. pertussis* BP338, $\:\varDelta\:$*lgmA-D* and the strains PagP_Ec_ + LpxE_Ft_, PagP_Ec_ / LpxE_Ft_, PagP_Ec_, LpxM_Ec_, PagP_Bp_ and LpxE_Ft_ in BP338 and $\:\varDelta\:$*lgmA-D* (**A**) and the strains PagL_Bb_ + PagP_Ec_, PagL_Bb_ + PagP_Bp_ and PagL_Bb_ in both BP338 and $\:\varDelta\:$*lgmA-D* (**B**) and purified LOS extracted from the same strains (**C** and **D**). Data in C and D were normalized to stimulation with either BP338 or $\:\varDelta\:$*lgmA-D* and expressed as mean percentage of NFκB activation +/- standard deviation. Graph shows the results of at least 3 independent experiments, each done in triplicate or quadruplicate, *n* = 9–15. Results for heat-killed cells (**A** and **B**) are represented in black (BP338) and white ($\:\varDelta\:$*lgmA-D*). For the LOS response (**C** and **D**), constructs that showed reduced endotoxicity are represented in red (BP338) and blue ($\:\varDelta\:$*lgmA-D*). One-way ANOVA (Brown-Forsythe and Welch ANOVA tests) was used for statistical analysis. p values: < 0.0001 (****), < 0.001 (***), < 0.01 (**), < 0.1 (*), no significant difference (ns). Error bars represent the SD of the mean

As a screen for the endotoxic potential of different *B. pertussis* strains, we employed the hTLR4 stimulation assay. We tested their ability to activate NFκB using HEK-Blue hTLR4 cells, which express CD14, TLR4, and MD-2 LPS receptors [23]. Examined strains included the MPLA-like variant (PagP_Ec_ + LpxE_Ft_), hexa-acylated variants (PagP_Ec_, LpxM_Ec_ and PagP_Bp_), penta-acylated variants (LpxE_Ft_, PagL_Bb_ + PagP_EC_ and PagL_Bb_ + PagP_Bp_) and tetra-acylated variant (PagL_Bb_) in either BP338 or $\:\varDelta\:$*lgmA-D* background. *E. coli* K12 and *B. pertussis* 18–323 were used as positive and negative controls [40]. 18–323 LOS is known to induce less TLR4 stimulation than BP338 due to the lack of GlcN modification on lipid A and possessing a shorter 3’ acyl chain [28]. All tested strains exhibited higher NFκB activation compared to BP338 or $\:\varDelta\:$*lgmA-D* (Fig. 5A and B). Since all lipid A modifications were reliant on the enhanced expression of foreign genes introduced through plasmids, the augmented stimulation from heat-killed cells in HEK-Blue hTLR4 assay could be attributed to the release of a greater number of LOS (LPS) molecules rather than the specific structure of lipid A moieties or the discharge of cellular components and “damage-associated molecular patterns” (DAMPs) that engage TLR4 receptors alongside LPS [31, 41]. To overcome this possibility, we stimulated hTLR4 cells with purified LOS from these variants. MPLA and LPS-EB from *E. coli* 0111:B4 (S-LPS) were used as controls. LOS from the MPLA-like variant (PagP_Ec_ + LpxE_Ft_) exhibited lower NFκB activation than BP338 (Fig. 5C), while all hexa-acylated variants (PagP_Ec_, LpxM_Ec_ and PagP_Bp_) induced higher NFκB activation. Penta-acylated variants with a single phosphate group (LpxE_Ft_) (Fig. 5C) as well as those with different lengths and arrangements of acyl chains (PagL_Bb_ + PagP_EC_ and PagL_Bb_ + PagP_Bp_), displayed lower NFκB induction, as did the tetra-acylated variant (PagL_Bb_) (Fig. 5D). Results were consistent for LOS variants in both BP338 and $\:\varDelta\:$*lgmA-D* backgrounds. To summarize, LOS variants of strains PagP_Ec_ + LpxE_Ft_, LpxE_Ft_, PagL_Bb_ + PagP_EC_, PagL_Bb_ + PagP_Bp_, and PagL_Bb_ exhibited reduced endotoxicity profiles compared to their respective parental strains.

### Activation of MyD88 and TRIF pathways in human macrophages

To expand on our NFκB activation findings, we assessed the ability of purified LOS from various *B. pertussis* strains to induce the release of MyD88 pathway cytokines (TNF-α and IL-6) and TRIF pathway cytokines (IP-10, MCP-1, and IFN-β) from human THP-1 derived macrophages. Using different LOS concentrations (10 µg/ml, 1 µg/ml, and 100 ng/ml), we monitored cytokine profiles via ELISA after 4 and 24 h of stimulation.

**Fig. 6:**
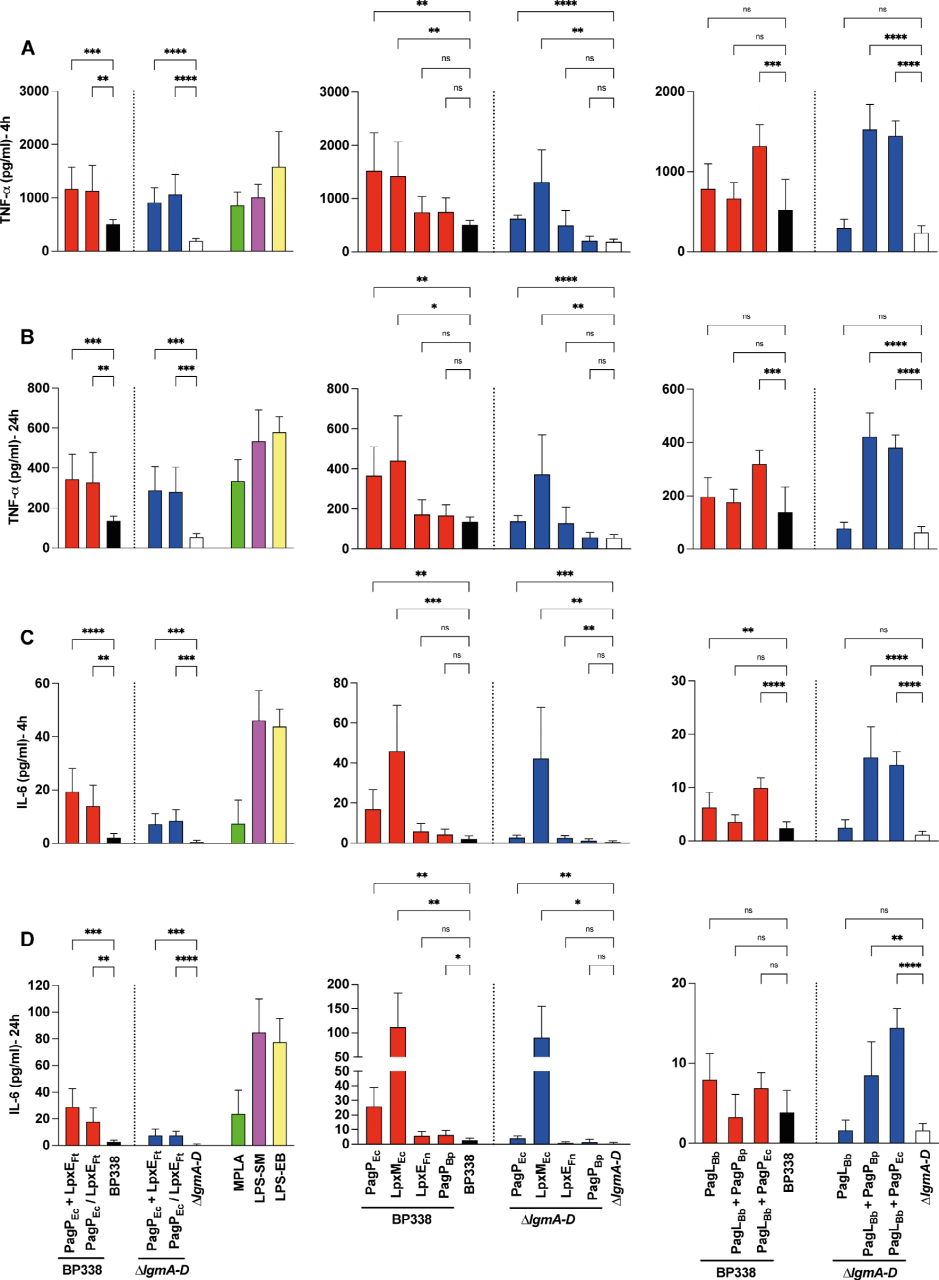
Release of MyD88-dependent pathway cytokines. Secreted cytokines/chemokines upon stimulation of THP-1-derived macrophages with 1𝜇𝑔/𝑚𝑙 of LOS extracted from *B. pertussis* BP338, $\:\varDelta\:$*lgmA-D* and the strains PagP_Ec_ + LpxE_Ft_, PagP_Ec_ / LpxE_Ft_, PagP_Ec_, LpxM_Ec_, LpxE_Ft_, PagP_Bp_, PagL_Bb_, PagL_Bb_ + PagP_Bp_, PagL_Bb_ + PagP_Ec_ in BP338 and $\:\varDelta\:$*lgmA-D*, measured by ELISA at 4 h and 24 h. (**A**) TNF-𝛼 – 4 h, (**B**) TNF-𝛼 – 24 h, (**C**) IL-6–4 h and (**D**) IL-6–24 h. Constructs made in BP338 WT are represented in red, while those in the $\:\varDelta\:$*lgmA-D* are in blue. BP338 controls are shown in black, and $\:\varDelta\:$*lgmA-D* controls are in white. One-way ANOVA (Brown-Forsythe and Welch ANOVA tests) was used for statistical analysis, *n* = 6–8. p values: < 0.0001 (****), < 0.001 (***), < 0.01 (**), < 0.1 (*), no significant difference (ns). Error bars represent the SD of the mean

**Fig. 7:**
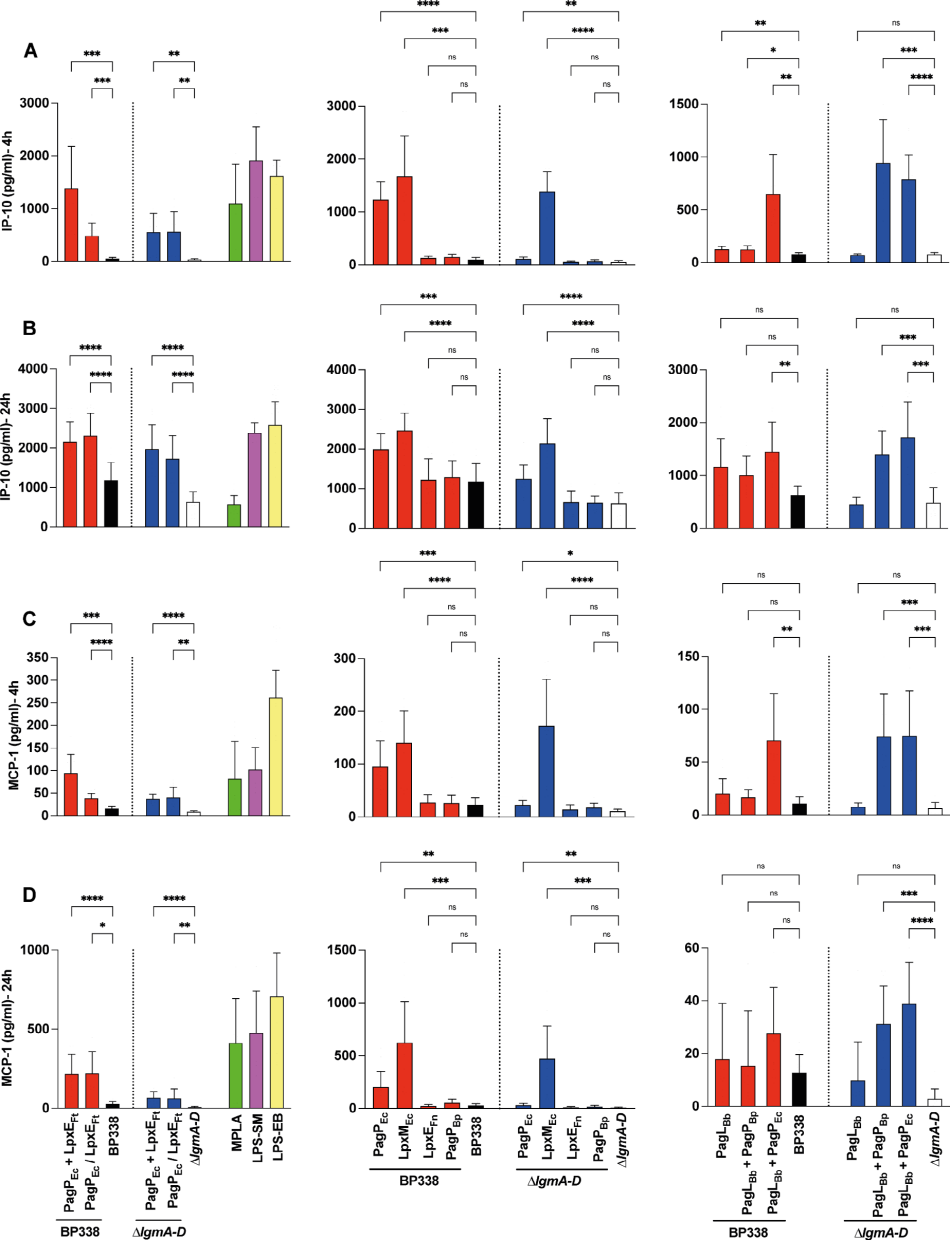
Release of TRIF-dependent pathway cytokines. Secreted cytokines/chemokines upon stimulation of THP-1-derived macrophages with 1𝜇𝑔/𝑚𝑙 of LOS extracted from *B. pertussis* BP338, $\:\varDelta\:$*lgmA-D* and the strains PagP_Ec_ + LpxE_Ft_, PagP_Ec_ / LpxE_Ft_, PagP_Ec_, LpxM_Ec_, LpxE_Ft_, PagP_Bp_, PagL_Bb_, PagL_Bb_ + PagP_Bp_, PagL_Bb_ + PagP_Ec_ in BP338 and $\:\varDelta\:$*lgmA-D*, measured by ELISA at 4 h and 24 h. (**A**) IP-10–4 h, (**B**) IP-10–24 h, (**C**) MCP-1–4 h and (**D**) MCP-1–24 h. Constructs made in BP338 WT are represented in red, while those in the $\:\varDelta\:$*lgmA-D* are in blue. BP338 controls are shown in black, and $\:\varDelta\:$*lgmA-D* controls are in white. One-way ANOVA (Brown-Forsythe and Welch ANOVA tests) was used for statistical analysis, *n* = 6–8. p values: < 0.0001 (****), < 0.001 (***), < 0.01 (**), < 0.1 (*), no significant difference (ns). Error bars represent the SD of the mean

Consistently, we observed an increase in both MyD88 and TRIF cytokines for the strains PagP_Ec_ + LpxE_Ft_, PagP_Ec_ / LpxE_Ft_, PagP_Ec_, LpxM_Ec_, and PagL_Bb_ + PagP_Ec_ in the BP338 background mostly in a dose-dependent manner at both time points. In the ∆*lgmA-D* background, the cytokine profiles of the same variants were similar, with an additional increase in cytokine release from PagL_Bb_ + PagP_Bp_ (Figs. 6 and 7 and Fig. S3 and S4).

Slight changes in lipid A acyl chain length can significantly affect its binding to the TLR4 complex and downstream signaling pathways. In *B. pertussis*, shortening the C3’ acyl chain reduces TLR4 stimulation [40, 42]. Despite both PagP_Bp_ and LpxM_Ec_ adding a secondary acyl chain to the C3’ position, they exhibit different substrate specificities. LpxM_Ec_ adds a myristate (C14) group, while PagP adds a palmitate (C16) group [31, 33]. We found that LOS from *B. pertussis* expressing LpxM_Ec_, having a hexa-acylated lipid A, showed much prominent immunostimulant activity compared to PagP_BP_, showing a higher level of release of all tested cytokines.

Comparing the cytokine response of our LOS from different strains to MPLA, LPS-SM, and LPS-EB, our MPLA-like structure (PagP_Ec_ + LpxE_Ft_) exhibited a profile similar to MPLA for all tested cytokines (Figs. 6 and 7), confirming its potential adjuvant properties. LOS molecules with increased cytokine profiles exhibited similar behaviour to MPLA and LPS-SM. Since we used LOS molecules in THP-1 derived macrophages stimulation, it is more appropriate to compare the cytokine profile to LPS-SM rather than MPLA. Notably, the response of MPLA, LPS-SM, and LPS-EB was primarily dose-independent, a characteristic shared by some of our strains, indicating that the concentrations used were sufficient for receptor saturation.

**Fig. 8:**
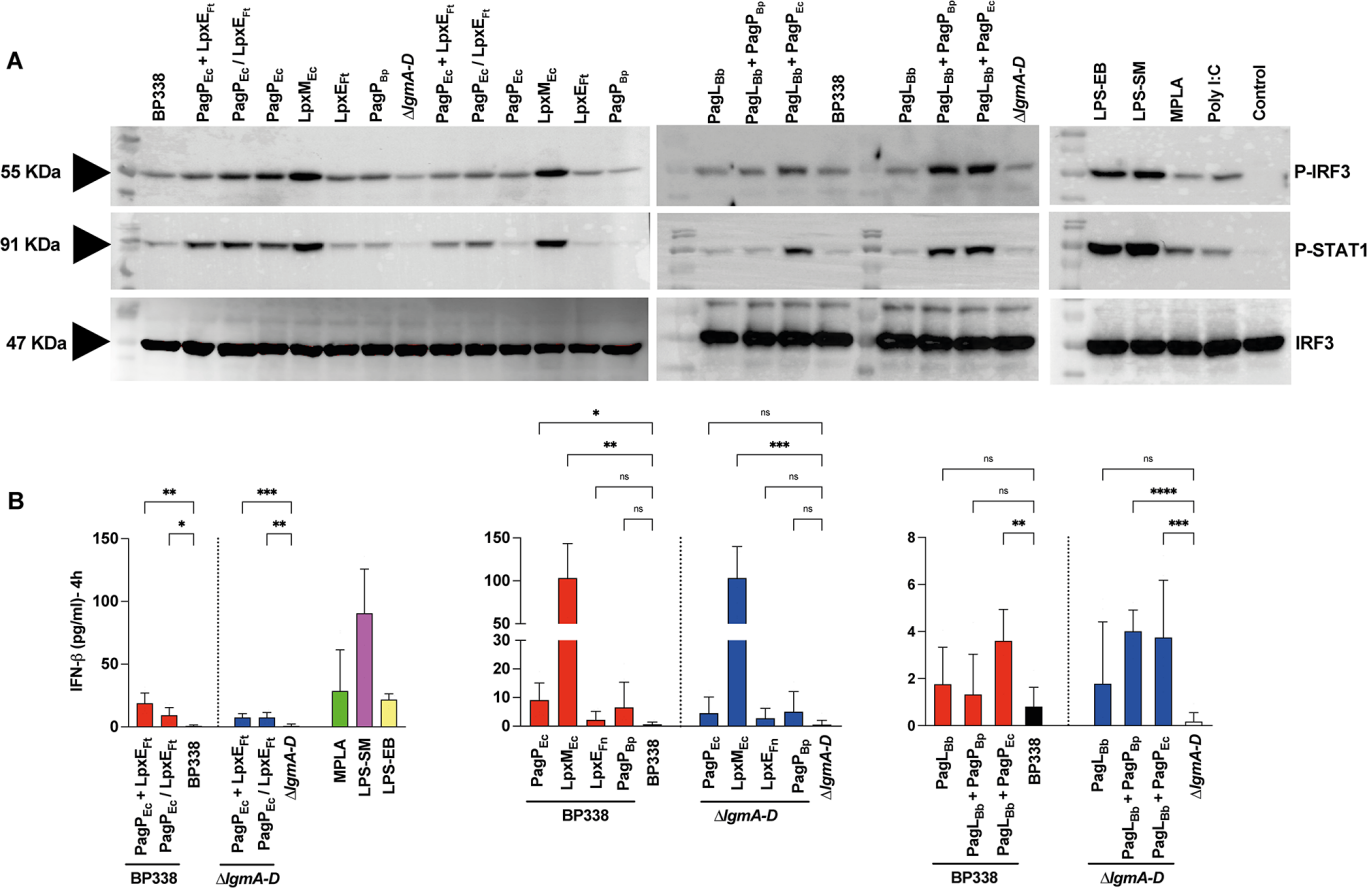
Activation of TRIF pathway in human macrophages. THP-1-derived Macrophages were treated with 1 𝜇𝑔/𝑚𝑙 of LOS extracted from indicated constructs. The levels of phospho-IRF3, phospho-STAT1, and IRF3 were detected by immunoblotting. Results are from one of three representative experiments (**A**). Secreted IFN-𝛽 upon stimulation of THP-1-derived macrophages with 1𝜇𝑔/𝑚𝑙 of LOS of *B. pertussis* BP338, $\:\varDelta\:$*lgmA-D* and the strains PagP_Ec_ + LpxE_Ft_, PagP_Ec_ / LpxE_Ft_, PagP_Ec_, LpxM_Ec_, LpxE_Ft_, PagP_Bp_, PagL_Bb_, PagL_Bb_ + PagP_Bp_, PagL_Bb_ + PagP_Ec_ in BP338 and $\:\varDelta\:$*lgmA-D*, measured by ELISA at 4 h (**B**). Constructs made in BP338 WT are represented in red, while those in the $\:\varDelta\:$*lgmA-D* are in blue. BP338 controls are shown in black, and $\:\varDelta\:$*lgmA-D* controls are in white. One-way ANOVA (Brown-Forsythe and Welch ANOVA tests) was used for statistical analysis, *n* = 6. p values: < 0.0001 (****), < 0.001 (***), < 0.01 (**), < 0.1 (*), no significant difference (ns). Error bars represent the SD of the mean

At 4 h post stimulation, we assessed IRF3 phosphorylation through western blot analysis. The purified LOS from strains PagP_Ec_ + LpxE_Ft_, PagP_Ec_ / LpxE_Ft_, PagP_Ec_, LpxM_Ec_, and PagL_Bb_ + PagP_Ec_ exhibited higher IRF3 phosphorylation compared to BP338 (Fig. 8A). In the $\:\varDelta\:$*lgmA-D* background, LOS from strains PagP_Ec_ + LpxE_Ft_, PagP_Ec_ / LpxE_Ft_, LpxM_Ec_, PagL_Bb_ + PagP_Ec_ and PagL_Bb_ + PagP_Bp_ showed a similar response (Fig. 8A). We then analyzed IFN-β induction through ELISA to see if it correlated with IRF3 activation. Consistent with the earlier findings, the LOS from strains that displayed enhanced IRF3 phosphorylation also exhibited increased IFN-β release, except for PagP_Ec_, which did not demonstrate a significant increase in IFN-β release compared to BP338 (Fig. 8B).

Because IFN-β release was generally limited, we also examined downstream STAT1 phosphorylation. Type I interferons (IFN-α and IFN-β) play an important role in innate viral immunity [19] and activate target genes through the JAK/STAT signaling pathway. Phosphorylated STAT1 translocates to the nucleus, binds to IFN-stimulated response elements (ISREs), and activates IFN-stimulated genes (ISGs) [43]. Poly I: C (Polyinosinic-polycytidylic acid), a potent inducer of type I IFN [19], was included as a control in the phosphorylation experiments along with MPLA, LPS-SM and LPS-EB. STAT1 phosphorylation levels followed a pattern similar to IRF3, suggesting that the release of IFN-β by our strains and controls was sufficient to induce ISGs (Fig. 8A). Our findings indicate that our LOS variants in strains PagP_Ec_ + LpxE_Ft_, PagP_Ec_ / LpxE_Ft_, PagP_Ec_, LpxE_Ft_, LpxM_Ec_, PagL_Bb_ + PagP_EC_ and PagL_Bb_ + PagP_Bp_ activated both the MyD88- and the TRIF-dependent pathways in THP-1 cells. The response of LOS of these strains was similar to that of LPS-SM and MPLA. Despite the robust NFκB activation observed in response to heat-killed cells from these strains (Fig. 5A and B), they exhibited a similar IRF3 and STAT1 phosphorylation pattern as their respective LOS counterparts (Fig. S5) suggesting their predisposition towards the TRIF pathway. It is noteworthy that effective adjuvants ideally stimulate both the MyD88 and TRIF pathways in a controlled level [18, 44]. Consequently, these candidates hold potential utility as vaccine adjuvants.

### Assessment of pyrogenicity by Monocyte Activation Test (MAT)

To assess the potential pyrogenicity of our candidates, we employed the Monocyte Activation Test (MAT), an in-vitro assay designed to detect the release of proinflammatory cytokines by human blood monocytes upon exposure to pyrogenic substances. The MAT has been endorsed by the European Pharmacopeia as an alternative to the Rabbit Pyrogen Test (RPT) following validation specific to the product [45]. Our focus was on measuring IL-6 release by human blood monocytes upon LOS stimulation, as IL-6 is a recognized marker for fever, inducing the production of prostaglandin E2 (PGE2) in the brain, triggering the febrile response [46].

We assessed the pyrogenicity of the LOS from the strains that were confirmed to activate the TRIF pathway (PagP_Ec_ + LpxE_Ft_, LpxE_Ft_, PagL_Bb_ + PagP_EC_, PagL_Bb_ + PagP_Bp_, PagP_Ec_, LpxM_Ec_) using the MAT. To quantify the pyrogenicity of our samples in EU/ml, we generated an endotoxin standard curve. Peripheral blood mononuclear cells (PBMC) were either untreated (blank) or stimulated with seven doses of reference standard endotoxin (RSE) ranging from 1.28 to 0.02 EU/ml, following European Pharmacopeia requirements. For PBMC stimulation, LOS samples were used at three dilutions (10 ng/ml, 5 ng/ml, and 2.5 ng/ml). These dilutions were carefully determined during preparatory testing to ensure detection of potential contaminants without exceeding the maximum valid dilution (MVD) for the product. IL-6 release into culture supernatants was measured via ELISA and results expressed in EU/ml, with a pyrogenicity limit of detection (LOD) at 0.04 EU/ml and a cut-off value of 0.025. Results were determined using the Reference Lot Comparison Test (MAT Method C) per the European Pharmacopeia. Concentration ratios were calculated by summing the mean concentration (EU/ml) of the three dilutions of the sample and dividing it by the sum of the mean concentration of the three dilutions of the reference lot (either BP338 or ∆*lgmA-D* in our study). The acceptance criterion was set as a ratio below 0.7, indicating a response 30% lower than the reference and considered non-reactogenic. Additionally, an OD ratio was calculated for each sample.

**Fig. 9:**
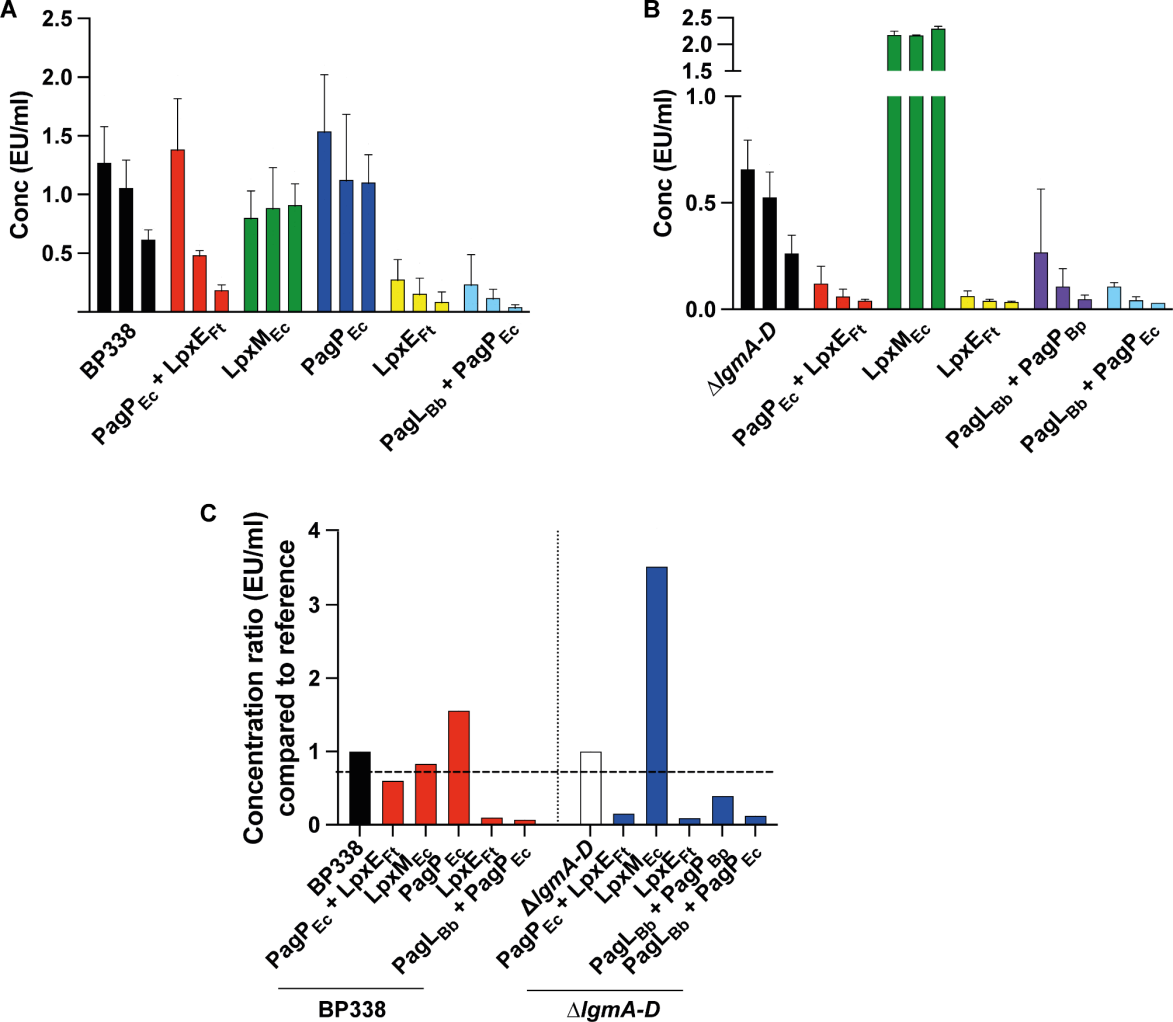
MAT assay. PBMC were stimulated with RSE and different concentrations (10, 5 and 2.5 ng/ml) of purified LOS. Release of IL-6 in terms of EU/ml upon stimulation of PBMC with LOS extracted from the strains PagP_Ec_ + LpxE_Ft_, LpxM_Ec_, PagP_Ec_, LpxE_Ft_, and PagL_Bb_ + PagP_Ec_ in BP338 (**A**) and strains PagP_Ec_ + LpxE_Ft_, LpxM_Ec_, LpxE_Ft_, PagL_Bb_ + PagP_Bp_ and PagL_Bb_ + PagP_Ec_ in $\:\varDelta\:$*lgmA-D* (**B**). The three columns per strain represent the three tested LOS concentrations from left to right (10 to 2.5 ng/ml). A concentration ratio was calculated which represents the sum of the mean concentration (EU/ml) of the 3 dilutions of the tested sample divided by the sum of the mean concentration of the 3 dilutions of the reference lot, which is either BP338 or Δ*lgmA-D* (**C**). The horizontal dashed line is the cut-off value for the assay. Error bars represent the SD of the mean

Our findings reveal that LOS from strains containing MPLA-like lipid A (PagP_Ec_ + LpxE_Ft_) and those with penta-acylated monophosphorylated lipid A (LpxE_Ft_), as well as varying acyl chain arrangements and lengths (PagL_Bb_ + PagP_EC_ and PagL_Bb_ + PagP_Bp_), exhibited markedly reduced potential to induce inflammatory cytokines in human PBMC compared to the unmodified penta-acylated lipid A found in the BP338 strain and ∆*lgmA-D* strain (Fig. 9). Notably, expressing the lipid A modifying enzymes in these variants within the GlcN mutant background reduced reactogenicity to a higher extent compared to their expression in the wild-type *B. pertussis* BP338 strain.

PagP_Ec_, containing hexa-acylated lipid A, resulted in elevated IL-6 production compared to BP338. However, when PagP_Ec_ was coupled with LpxE_Ft_, forming a hexa-acylated monophosphorylated lipid A (PagP_Ec_ + LpxE_Ft_), the triggered IL-6 response was notably lower, around 40% less than BP338. Furthermore, IL-6 production dropped over 80% with LpxE_Ft_ and PagL_Bb_ + PagP_EC_ (Fig. 9A and C). Notably, PagL_Bb_ + PagP_Bp_ displayed nearly a 60% lower IL-6 response compared to $\:\varDelta\:$*lgmA-D*. Using OD values instead of interpolated concentrations yielded consistent results (Fig. S6). These findings suggest that LOS harboring these lipid A modifications are less likely to elicit side effects such as fever.

In our study, our primary focus was the modification of *B. pertussis* BP338’s lipid A components, while keeping other antigenic components unchanged. We assessed the impact of these lipid A changes on the expression of virulence factors, BrkA and TcfA, critical for serum resistance and host colonization [47, 48] and found that their expression levels in our modified strains matched those of BP338 (Fig. S7A and B). Additionally, we examined the overall protein profile of the different strains using Page Blue staining of whole cell lysates, and all strains exhibited identical patterns (data not shown). These findings suggest that most wild-type membrane proteins and virulence factors remain unaffected by our lipid A modifications and would thus elicit suitable immune responses.

## Discussion

The interplay between lipid A and TLR4, offers a strategic avenue for modulating immune reactions. By harnessing this mechanism, adjuvants that orchestrate finely tuned immune responses can be engineered, thereby enhancing the efficacy of vaccines. MPLA, derived from *Salmonella minnesota* R595, is a widely recognized vaccine adjuvant [25] known for its ability to induce a robust Th1 immune response and guide signaling through the TRIF pathway, ensuring sufficient adaptive immune activation without excessive release of proinflammatory cytokines [18, 24]. Maintaining a regulated level of activation in the MyD88 and TRIF pathways is beneficial for establishing long-lasting immunity in vaccines [25].

The use of MPLA as an adjuvant is expensive, as it requires a complex chemical synthesis process involving 24 steps [44]. In this study, our primary objective was to develop an MPLA-like adjuvant strain in *B. pertussis*. We employed a systematic approach by engineering various *B. pertussis* strains, each featuring distinct arrays of lipid A modifications. We created a hexa-acylated monophosphorylated lipid A molecule that significantly reduced the endotoxic potential while favoring activation of the TRIF pathway – akin to MPLA. Consequently, the engineered strain induced the activation of IRF3 phosphorylation and its LOS showed a marked reduction in the IL-6 response when tested in PBMCs. These findings closely mirrored the responses typically observed with MPLA suggesting that our engineered hexa-acylated monophosphorylated lipid A molecule could function as an adjuvant. Furthermore, our research yielded additional variants, aside from MPLA, that demonstrated reduced endotoxicity and heightened adjuvanticity. Most significantly, our study effectively addressed the reactogenicity associated with *B. pertussis* LOS. It’s worth noting that the absence of GlcN modification represents an additional factor, alongside the other modifying enzymes, contributing to the reduction in reactogenicity.

Needham et al. pioneered a combinatorial lipid A modification approach for immune modulation. By employing synthetic lipid biology in *E. coli*, they created diverse lipid A molecules, yielding various TLR4 responses for therapeutic adaptability [24]. Their research showed that combining lipid A-modifying enzymes from different bacteria is more effective than using them individually. They also showed that the addition of *E. coli’*s PagP alongside phosphatases such as LpxE to a pentaacylated background strain resulted in a hexaacylated structure with increased endotoxicity. Unexpectedly, adding the LpxE phosphatase actually increased endotoxicity, which challenged the notion that phosphate group removal reduces TLR4 interaction [24]. We also utilized PagP to promote hexa-acylated lipid A formation, pairing it with LpxE or PagL. The removal of the 1-phosphate by LpxE from hexa-acylated LOS significantly reduced TLR4-MD2-CD14 pathway stimulation, and deacylation by PagL lessened the endotoxic activity as expected. Our findings underscore the importance of empirically determining the optimal combination of modifying enzymes for different bacterial species to engineer controlled lipid A modifications that yield diverse responses.

It’s worth noting that the expression of various lipid A modifying enzymes in bacteria often results in a mixed array of lipid A species, as seen in our study with multiple strains expressing different enzymes. However, this diversity doesn’t diminish the significance of our approach, as MPLA itself encompasses various forms with acyl chain counts ranging from 3 to 6 [49].

Our results also indicated that the IL-6 response in PBMCs varied when compared to the stimulation of THP-1 derived macrophages with the same LOS samples. While the behavior of cytokine release differed, the overall response was relatively weak in THP-1 cells. This could be attributed to the lower expression of CD14 in THP-1 cells compared to the higher levels found in PBMCs, which are responsible for significant LPS responsiveness. Consequently, it appears that THP-1, a spontaneously immortalized leukemic cell line with defined monocytic markers, may have limitations as a model for primary monocytes [50].

Recent efforts have been made to enhance pertussis vaccines [51–54]. Our unique approach adjusts *B. pertussis* LOS to create an adjuvant-like strain akin to MPLA, potentially serving as a basis for an improved wP vaccine or as an adjuvant to enhance aP vaccines. The heat-killed strains with these lipid A modifications stimulated higher NFκB levels; however, their activation of the TRIF pathway makes them promising candidates for further study as next-generation wP pertussis vaccines. Optimization of NFκB activation would need to be achieved, possibly by further controlling expression of the lipid A modifying enzymes.

Utilizing the natural biosynthetic pathways of *B. pertussis* offers a cost-effective adjuvant solution by eliminating the need for using MPLA. In our research, we successfully manipulated the lipid A of *B. pertussis* and suggest that these lipid A modifications are unlikely to induce adverse reactions in humans. We propose four promising adjuvants with varied lipid A structures, including hexa-acylated mono-phosphorylated MPLA-like (PagP_Ec_ + LpxE_Ft_), penta-acylated monophosphorylated (LpxE_Ft_), and penta-acylated variants with alterations in acyl chain position and number (PagL_Bb_ + PagP_EC_ and PagL_Bb_ + PagP_Bp_). Indeed, given that aP vaccine antigens are purified from *B. pertussis*, the LOS from these strains can similarly undergo purification and be incorporated in formulations for future aP vaccines to overcome the limitations of these vaccines.

## Materials and methods

### Strains and growth conditions

All strains and plasmids used in this work are listed in Table S1. *B. pertussis* strains were allowed to grow on Bordet-Gengou (BG) agar (BD Biosciences) supplemented with 15% sheep blood (Dalynn) at 37 °C for 72 h. Bacteria from solid medium were used to inoculate complete Stainer-Scholte (SS) broth [55] containing 0.15% bovine serum albumin; BSA (Sigma) [56] and SS-supplements to give an optical density at 600 nm of 0.01 and incubated under agitation till reaching mid-log phase or stationary phase. Bacteria were harvested into phosphate-buffered saline (PBS), and killed by incubation for 60 min at 56 °C [28].

For all the strains used, media were supplemented with nalidixic acid (30 µg/mL). When required, for selection or plasmid maintenance, media were supplemented with kanamycin (100 µg/ml), gentamicin (30 µg/mL), and 100 ng/ml anhydrotetracycline (Cayman Chemical) to induce protein expression in *B. pertussis* using the pIG10 plasmid (Ifill and Fernandez, manuscript in preparation). *E. coli* strains DH5$\:\alpha\:$ (Invitrogen) and RHO3 were used for cloning and as the donor strain for diparental mating, respectively. Both strains were grown at 37 °C in Luria-Bertani (LB) broth or on LB agar. RHO3 was grown in medium supplemented with 2,6-diaminopimelic acid (DAP) (250 mg/ml). Antibiotics were purchased from Sigma and Thermo Scientific.

### Genetic manipulations

Recombinant DNA techniques were performed according to standard techniques. The primers used are listed in Table S2 and were synthesized by Integrated DNA Technologies. Genes encoding lipid A modifying enzymes were amplified by PCR using Q5 High-Fidelity DNA Polymerase. The genes used in this study are: *pagP*_*Bp*_, *B. pertussis*; *pagP*_*Ec*_, *E. coli*; *pagL*_*Bb*_, *B. bronchiseptica*; *lpxE*_*Ft*_, *Francisella tularensis*. PCR products were purified, cut by restriction enzymes and ligated to pBBR2Pcpn [28], pB4 (Jun and Fernandez, manuscript in preparation) or pIG10 by T4 DNA ligase. DNA polymerase, restriction enzymes and T4 DNA ligase were purchased from New England Biolabs (NEB, Canada). pIG10-PagP_Ec_/LpxE_Ft_ was constructed by FastCloning [57] using pIG10-LpxE_Ft_ as a template and Phusion DNA polymerase. The PCR product was purified and treated with DpnI. *E. coli* strain DH5α was transformed with the ligated product or DpnI treated PCR product using standard protocols [58]. The clones containing the target gene inserted into the corresponding plasmid were selected by colony PCR and confirmed by DNA sequencing. Plasmids were then transferred to RHO3 and subsequently to BP338 and BP338 ($\:\varDelta\:$*lgmA-D*) by diparental mating [59] using the appropriate antibiotics for counterselection.

### Lipid A extraction and mass spectrometry

Lipid A modifications were investigated by mass spectrometry (MS) analysis using Matrix-assisted laser desorption/ionization time of flight (MALDI-TOF). Cells were grown for 48 h in complete SS-medium supplemented with 0.15% BSA and antibiotics as appropriate. Cultures were centrifuged (10000×g), washed twice with phosphate buffer (10 mM Na2HPO4, 1.7 mM KH2PO4) and freeze-dried. Lipid A was extracted from lyophilized cells and de-salted as previously described [39]. Ten milligrams of the lyophilized bacterial cells were resuspended in 400 µl of a mixture of isobutyric acid: 1 M ammonium hydroxide (5:3, v/v) and kept on a heat block for 2 h at 100 °C with vortexing every 15 min, then centrifuged at 2000 × g for 15 min and the supernatant was mixed with a double volume of water and lyophilized overnight. Samples were then washed with methanol and lipid A was solubilized in 80 µL of chloroform: methanol: water (3:1.5:0.25, v/v). Lipid A suspension was desalted with a few grains of ion-exchange resin (Dowex 50 W-X8; H+). Two microliters aliquot of lipid A suspension was loaded on a polished steel target, air dried and covered by 1 µL of 2,5-dihydroxybenzoic acid matrix (Sigma) dissolved in 0.1 M citric acid solution in chloroform: methanol: water (3:1.5:0.25, v/v) and allowed to air dry. The target was inserted in Applied Biosystems MALDI-TOF spectrometer. Data acquisition and analysis were performed using the Data Explorer software.

### LOS extraction and purification

LOS was prepared from *B. pertussis* as previously described [60]. Briefly, bacterial pellets were resuspended in lysis buffer (2% sodium dodecyl sulphate (SDS), 4% β-mercaptoethanol (2-ME), and 0.5 M of Tris-HCl, pH 6.8) and boiled for 10 min. The lysed cells were treated with DNase, RNase and proteinase K. Crude extracts were treated with warm 90% phenol solution to extract all proteins. The aqueous phase was separated and treated with diethyl ether saturated with Tris-EDTA to remove residual phenol. LOS preparations were then precipitated by 75% ethanol. The precipitates were resuspended in 200 µL of 0.2% TEA in endotoxin-free water and kept at -20 °C. LOS preparations were quantified by the purpald assay [60–62].

### Cell cultures

HEK-Blue (InvivoGen) cell lines hTLR4 and Null2 were maintained in Dulbecco’s Modified Eagle’s Medium (DMEM) supplemented with 10% heat-inactivated fetal bovine serum, 2 mM GlutaMAX, 1 mM sodium pyruvate, 100 U/ml penicillin, 100 $\:\mu\:$g/ml streptomycin (GIBCO, Thermo Fisher) and 100ug/mL Normocin (InvivoGen). hTLR4 cells were grown in the presence of Zyocin (100 ug/mL), Hygrogold (100 ug/mL) and Blasticidin (10 ug/mL) for selection while Null2 cells were grown in the presence of Zyocin only [28].

THP-1 cells (ATCC) were maintained in RPMI 1640 medium supplemented with 10% heat-inactivated fetal bovine serum, 2 mM GlutaMAX, 1 mM sodium pyruvate, 50 U/ml penicillin and 50 $\:\mu\:$g/ml streptomycin [40]. Human peripheral blood mononuclear cells, PBMC (Lonza) were maintained in Iscove’s Modified Dulbecco Medium (IMDM) with added 5% human serum supplement [63]. Cells were incubated at 37 °C in humid air with 5% CO_2_.

### HEK-Blue TLR4 activation assay

The level of endotoxicity of different *B. pertussis* constructs was assessed according to manufacturer’s guidelines [28]. Briefly, HEK-Blue hTLR4 and Null2 were stimulated with 100 ng/ml of purified LOS or heat-killed *B. pertussis* cells. All LOS and control LPS stock solutions were sonicated for 10 min each time immediately before use. After 20–24 h of incubation, the supernatants were assayed for secreted embryonic alkaline phosphatase (SEAP) induced by NFκB by using QUANTI-Blue (InvivoGen) reagent. Absorbance at 650 nm was used as a readout. Null2 cell line was used as negative control.

### THP-1 stimulation and cytokine detection

THP-1 cells were differentiated into macrophages by incubation with 50 ng/ml phorbol 12-myristate 13-acetate, PMA (Sigma-Aldrich) for 48 h [23]. PMA was then removed, and the cells were washed and rested for another 72 h. Stimulation of THP-1 derived macrophages was performed by incubating the cells with cell culture medium supplemented with purified LOS extracted from different *B. pertussis* constructs in 3 different concentrations (10 µg/ml, 1 µg/ml and 100 ng/ml). MPLA from *S. minnesota*, LPS-EB; Smooth LPS from the Gram-negative *E. coli* 0111:B4 (S-LPS) and LPS-SM; Rough-LPS from *Salmonella minnesota* R595 (R-LPS) were used as controls and were purchased from InvivoGen. All LOS and control LPS stock solutions were sonicated for 10 min each time immediately before use.

### Enzyme-linked immunosorbent assay (ELISA)

To quantify the released cytokines, tissue culture supernatants were assayed using DuoSet ELISA kits (R&D systems) at 4 and 24 h. All assays were performed according to the manufacturers’ instructions. TNF-α, IL-6, IP-10, MCP-1 and IFN-β levels were measured in THP-1 derived macrophages supernatants after the indicated timing. The optical density at 450 nm was determined using a microplate reader (wavelength correction: 540 nm).

### Western blot analysis

Immunoblotting of IRF3, P-IRF3 and P-STAT1 was performed as described [44]. Stimulated THP-1 cells were washed in ice-cold PBS, after removal of supernatants, and lysed in radioimmunoprecipitation assay buffer (RIPA) buffer (Abcam). Cell extracts were centrifuged for 5 min at 12,000 g, the supernatant collected, and protein content was quantified by the Bradford assay. Cell supernatants were resuspended in the Laemmli buffer and denatured for 5 min at 100 °C. Protein separation was performed by 12% SDS-PAGE and transferred to polyvinylidene difluoride (PVDF) membranes, blocked in 5% w/v skim milk, and incubated with the primary and corresponding secondary antibodies. Proteins were revealed by chemiluminescence (GE Healthcare) and detected using the BioRad Imaging System. The PVDF membrane filters were incubated with the following primary antibodies: anti-phospho-IRF-3 (Ser386) rabbit mAb (Abcam), anti-phospho-STAT1 (Tyr701) rabbit mAb (Cell Signaling Technology), and anti-IRF3 rabbit mAb (Fitzgerald Industries). Polyclonal anti-rabbit HRP-linked IgG (Jackson ImmunoResearch) was used as the secondary antibody.

### Monocyte Activation Test

PyroCell Monocyte Activation Test (MAT) Kit was purchased from Lonza. The kit contains pooled PBMCs from 4 donors and PeliKine Compact human IL-6 ELISA Kit. Cell culture stimulation was performed according to Methods B and C as described in Ph. Eur. Chapter 2.6.30 (EDQM, 2017). Briefly, two 2-fold dilutions of purified LOS of *B. pertussis* vaccine candidates and Reference Standard Endotoxin (RSE) (Lonza) were performed directly in a flat-bottom 96-well cell culture plate (Costar) prior to seeding the stimuli in the 96-well plate. Quadruplicates of the LOS concentrations were prepared in the 96-well cell culture plate. Then, 100,000 cells were added to each well to a final volume of 200 µL. Culture plates were incubated at 37 °C for 22 h in a humidified atmosphere containing 5% CO2. After incubation of the cells, the supernatant from each well was recovered and cytokine determination was performed using the PeliKine compact human IL-6 ELISA kit (Lonza).

### Statistical analysis

All experimental results represent the mean ± standard deviation (SD) of at least three independent experiments unless specified. The western blots shown were representative data from at least two independent experiments. For ELISA experiments, means were compared by one-way ANOVA. All statistical analyses were performed using GraphPad Prism 9.

## Electronic supplementary material

Below is the link to the electronic supplementary material.

Supplementary Material 1

## Data Availability

Data is provided within the manuscript and supplementary information files.
