# Supplementary material for: Programming Bordetella pertussis lipid A to promote adjuvanticity
Source: Microb Cell Fact. 2024 Sep 14;23:250. doi: 10.1186/s12934-024-02518-7 (PMC11401268; doi:10.1186/s12934-024-02518-7)
Supplement: Supplementary file 1 — Supplementary Material 1 [file 12934_2024_2518_MOESM1_ESM.docx]

**Table S1. Bacterial strains and plasmids used in this study.**

| **Strain or plasmid** | **Description** | **Source or reference** |
| --- | --- | --- |
| ***B. pertussis* Strains** |  |  |
| BP338 | Wild-type *B. pertussis* Tohama I strain; Nal^R^ | A. Weiss [59] |
| 18-323 | Wild-type *B. pertussis* Tohama I strain | ATCC |
| BP338 $\Delta$*lgmA-D* | $\Delta$*lgmABCD* | [28] |
| BP347 | Isogenic BvgS mutant (*bvgS*::Tn5) | [60] |
| ***E. coli* Strains** |  |  |
| DH5-$\alpha$ | Molecular cloning strain | Invitrogen |
| RHO3 | Conjugation strain; Km^s^ $\Delta$asd $\Delta$aphA, DAP auxotroph | [61] |
| LpxE_Ft_-BP338/$\Delta$*lgmA-D* | BP338 or $\Delta$*lgmA-D* with pIG10-LpxE_Ft_ | This work |
| LpxM_Ec_-BP338/$\Delta$*lgmA-D* | BP338 or $\Delta$*lgmA-D* with pIG10-LpxM_Ec_ | This work |
| PagL_Bb_-BP338/$\Delta$*lgmA-D* | BP338 or $\Delta$*lgmA-D* with pIG10-PagL_Bb_ | This work |
| PagP_Bp_-BP338/$\Delta$*lgmA-D* | BP338 or $\Delta$*lgmA-D* with pBBR2pcpn-PagP_Bp_ | This work |
| PagP_Ec_-BP338/$\Delta$*lgmA-D* | BP338 or $\Delta$*lgmA-D* with pBBR2pcpn-PagP_Ec_ | This work |
| PagP_Ec_/LpxE_Ft_-BP338/$\Delta$*lgmA-D* | BP338 or $\Delta$*lgmA-D* with pIG10-PagP_Ec_/LpxE_Ft_ | This work |
| PagP_Ec_ + LpxE_Ft_-BP338/$\Delta$*lgmA-D* | BP338 or $\Delta$*lgmA-D* with pIG10-LpxE_Ft_ and pBBR2pcpn-PagP_Ec_ | This work |
| PagL_Bb_ + PagP_Bp_ -BP338/$\Delta$*lgmA-D* | BP338 or $\Delta$*lgmA-D* with pIG10-PagL_Bb_ and pBBR2pcpn-PagP_Bp_ | This work |
| PagL_Bb_ + PagP_Ec_ -BP338/$\Delta$*lgmA-D* | BP338 or $\Delta$*lgmA-D* with pIG10-PagL_Bb_ and pBBR2pcpn-PagP_Ec_ | This work |
|  |  |  |
| **Plasmids** |  |  |
| pBBR2pcpn | Broad-range, low copy number plasmid containing the Pcpn heat shock promoter adjacent to multiple cloning site; Kan^R^ | [28] |
| pSS4894 | Suicide vector containing I-SceI restriction enzyme and cognate restriction site, used for allelic exchange; Gm^R^ | [62] |
| pB4 | Low copy number plasmid containing the Pcpn heat shock promoter and 6x His at C-terminal; Kan^R^ | (Jun and Fernandez, manuscript in preparation) |
| pIG10 | Contains tet-inducible promoter; Gm^R^ | (Ifill and Fernandez, manuscript in preparation) |
| pIG10-LpxE_Ft_ | pIG10 containing *lpxE* of *Francisella tularensis* | This work |
| pIG10-LpxM_Ec_ | pIG10 containing *lpxM* of *E. coli* | This work |
| pIG10-PagL_Bb_ | pIG10 containing *pagL* of *B. bronchiseptica* | This work |
| pBBR2pcpn-PagP_Bp_ | pBBR2pcpn containing *pagP* of *B. pertussis* | This work |
| pBBR2pcpn-PagP_Ec_ | pBBR2pcpn containing *pagP* of *E. coli* | This work |
| pIG10-PagP_Ec_/LpxE_Ft_ | pIG10 containing both PagP of *E. coli* and *lpxE* of *Francisella tularensis* | This work |

**Table S2. Primers used in this study.**

| **Primer name** | **Primer sequence** |
| --- | --- |
| PagP_Bp_-EcoRI-For | ttttGAATTCATGCATCATCACCATCACCACACCCAGTATTTCCGGTCCCTG |
| PagP_Bp_-XbaI-Rev | ttttTCTAGATTAGAACTCCCAGCGGCCAAACAT |
| PagP_EC_-EcoRI-For | ttttGAATTCATGCATCATCACCATCACCACAACGTGAGTAAATATGTCGCT |
| PagP_EC_-XbaI-Rev | ttttTCTAGATCAAAACTGAAAGCGCATCCAGGC |
| PglL_Bb_-EcoRI-For | ttttttGAATTCATGCCGGCGGAAACCACCGTGTCGGGGG |
| PglL_Bb_-SalI-Rev | ttttttGTCGACTCATTTGCACGGCTTGGCCTCCGGATGC |
| LpxM_Ec_-EcoRI-For | ttttttGAATTCATGGAAACGAAAAAAAATAATAGCGAAT |
| LpxM_Ec_-SalI-Rev | ttttttGTCGACTTTGATGGGATAAAGATCTTTGCGCTT |
| PagP_Ec_-For (FastCloning) | GCAACCGCGAGATCATCTGAGAGGAGAAATTAACCATGAACGTGAGTAAATATGTCGC |
| PagP_Ec_-Rev (FastCloning) | TGACCACCACCGTCGACAAACTGAAAGCGCATCCAGGCAAAG |
| pIG10/LpxE_Ft_-Rev (FastCloning) | GATGATCTCGCGGTTGCGCATCCAGTAGTAGATG |
| pIG10/LpxE_Ft_-For (FastCloning) | GTCGACGGTGGTGGTCATCATCACCATCAC |

**Table S3. List of observed *m/z* ion peaks by MALDI-TOF analysis of LPS samples from *B. pertussis* BP338 parental and engineered strains used in this study.**

| **Observed ion *(m/z)*** | **Assignment** |
| --- | --- |
| **1333** | Tetra-acyl [2 GlcN + 2 OH-C14 + 1 OH-C10 + 1 C14], 2 P |
| **1559** | Penta-acyl [2 GlcN + 3 OH-C14 + 1 OH-C10 + 1 C14], 2 P |
| **1720** | Penta-acyl [2 GlcN + 3 OH-C14 + 1 OH-C10 + 1 C14], 1 GlcN, 2 P |
| **1798** | Hexa-acyl [2 GlcN + 3 OH-C14 + 1 OH-C10 + 1 C14 + 1 OH-C16], 2 P |
| **1959** | Hexa-acyl [2 GlcN + 3 OH-C14 + 1 OH-C10 + 1 C14 + 1 OH-C16], 1 GlcN, 2 P |
| **1770** | Hexa-acyl [2 GlcN + 4 OH-C14 + 1 OH-C10 + 1 C14], 2P |
| **1931** | Hexa-acyl [2 GlcN + 4 OH-C14 + 1 OH-C10 + 1 C14], 1 GlcN, 2 P |
| **1480** | Penta-acyl [2 GlcN + 3 OH-C14 + 1 OH-C10 + 1 C14], 1 P |
| **1391** | Tetra-acyl [2 GlcN + 2 OH-C14 + 1 C14], 2 P |
| **1165** | Tri-acyl [2 GlcN + 2 OH-C14 + 1 C14], 2 P |
| **1630** | Penta-acyl [2 GlcN + 2 OH-C14 + 1 OH-C16 + 1 C14], 2 P |

**Spectra corresponding to the ion peaks are shown in Fig. 3 and 4. The assignments were based on previously published data [23, 38]. GlcN, glucosamine; P, phosphate.**

**Supplementary Figures**

**
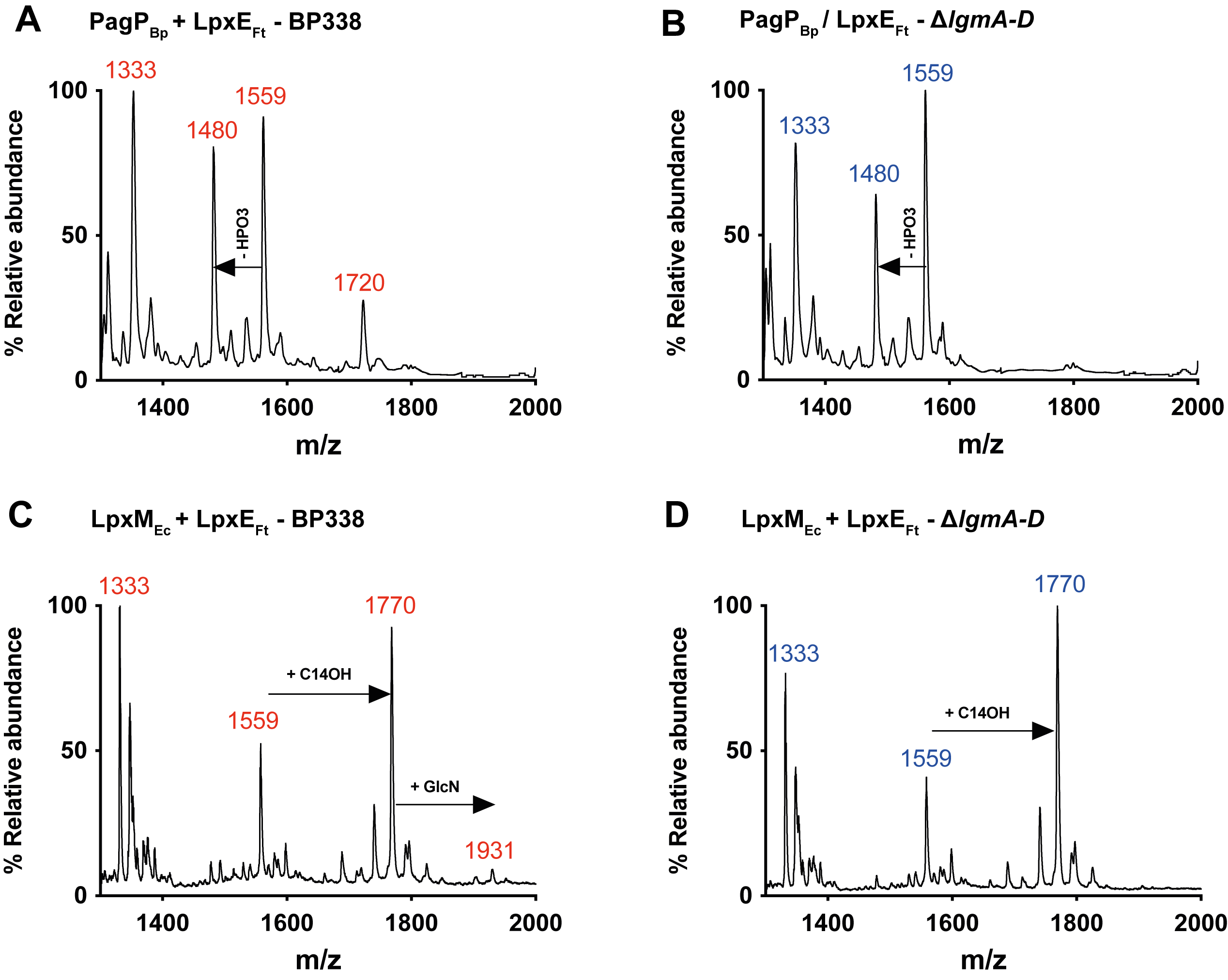
**

**Figure S1.** **Structural analysis of lipid A obtained from the incompatible combinations of PagP_Bp_ or LpxM_Ec_ with LpxE_Ft_ in** ***B. pertussis* BP338 engineered strains.**

Negative-ion MALDI-TOF mass spectra of lipid A isolated from *B. pertussis* BP338 strain PagP_Bp_ + LpxE_Ft_ (A and B) and LpxM_Ec_ + LpxE_Ft_ (C and D).

**
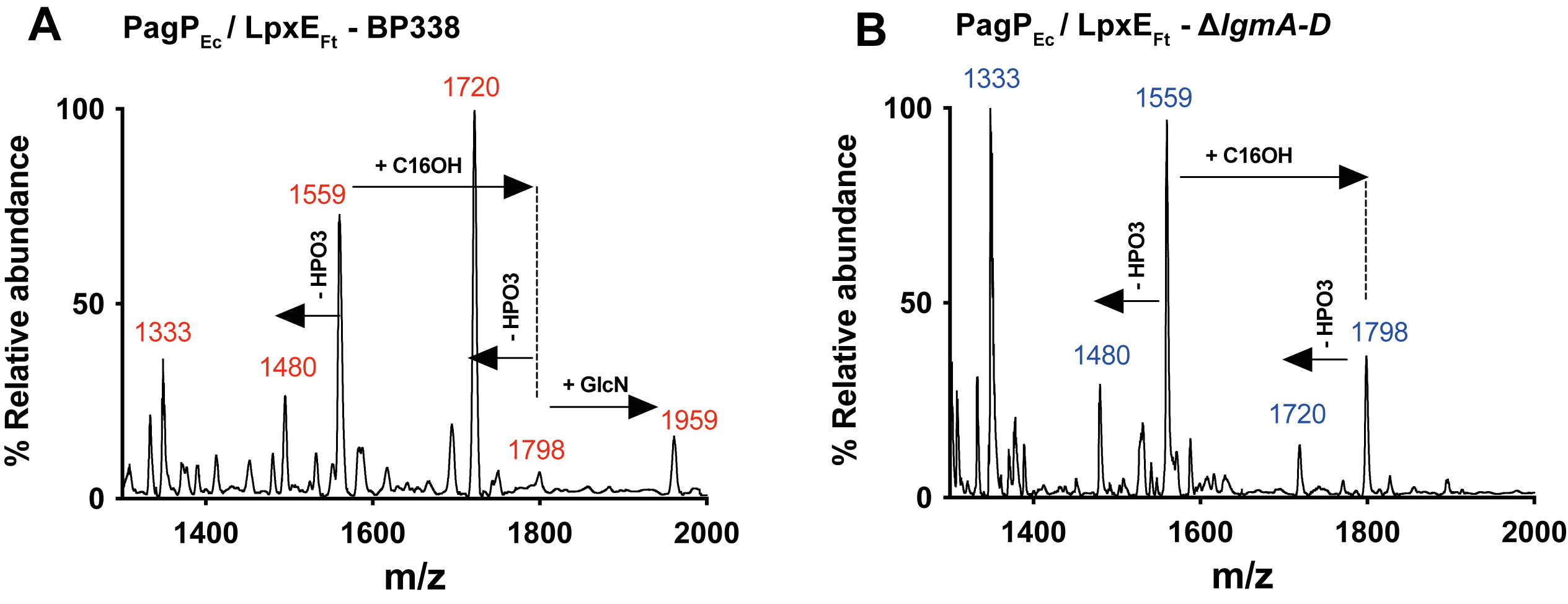
**

**Figure S2.** **Structural analysis of the monophosphorylated hexa-acylated lipid A in *B. pertussis* BP338 strain.**

Negative-ion MALDI-TOF mass spectra of lipid A isolated from *B. pertussis* BP338 strain PagP_Ec_ / LpxE_Ft_ expressing PagP_Ec_ and LpxE_Ft_ on the same plasmid (pIG10).

**
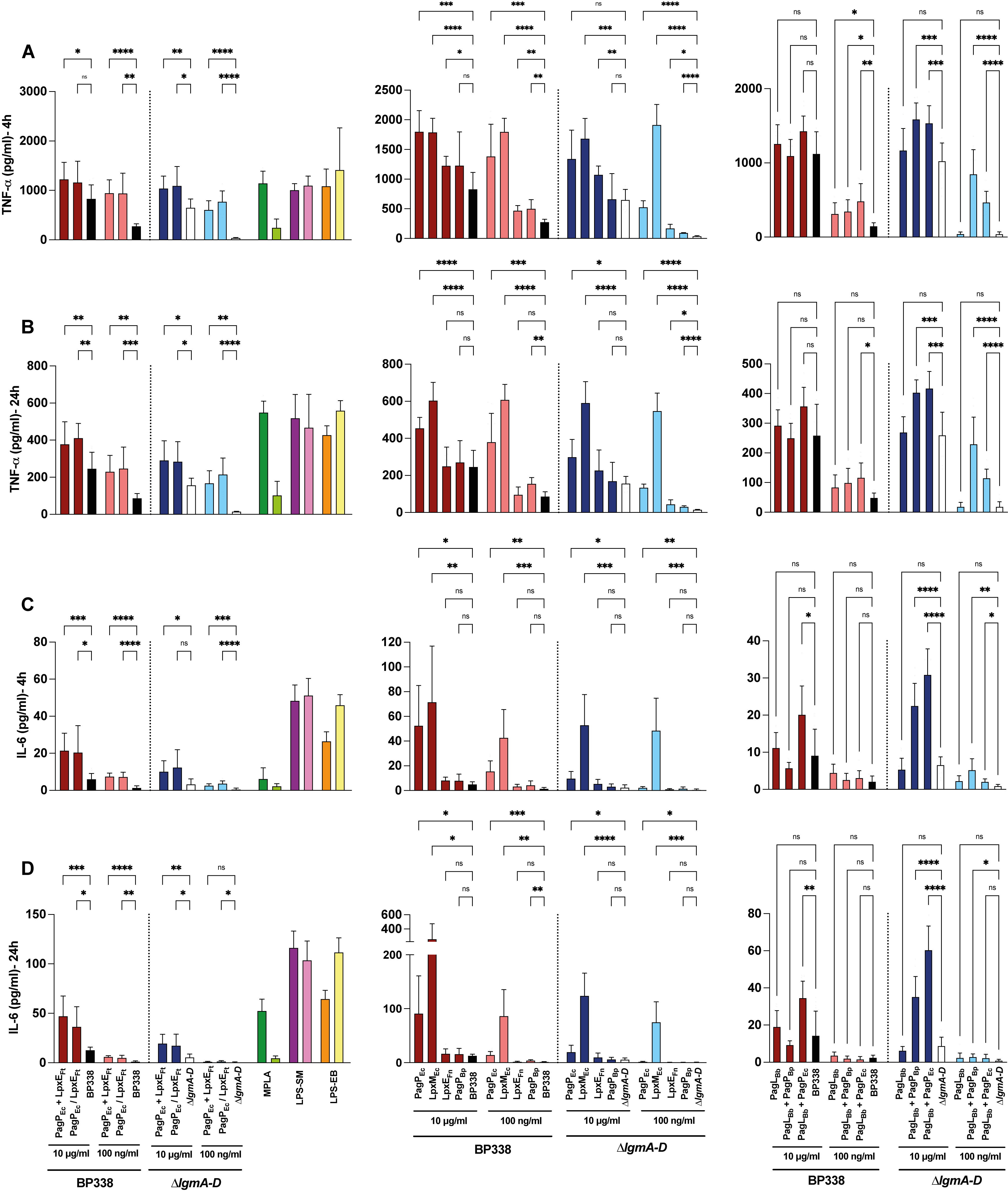
**

**Figure S3. Release of MyD88-dependent pathway cytokines.**

Secreted cytokines/chemokines upon stimulation of THP-1-derived macrophages with 2 different doses of LOS (10 𝜇𝑔/𝑚𝑙 and 100ng/ml) extracted from *B. pertussis* BP338, $\Delta$*lgmA-D* and the strains PagP_Ec_ + LpxE_Ft_, PagP_Ec_ / LpxE_Ft_, PagP_Ec_, LpxM_Ec_, LpxE_Ft_, PagP_Bp_, PagL_Bb_, PagL_Bb_ + PagP_Bp_, PagL_Bb_ + PagP_Ec_ in BP338 and $\Delta$*lgmA-D*, measured by ELISA at 4h and 24h. (A) TNF-𝛼 – 4 h, (B) TNF-𝛼 – 24 h, (C) IL-6 – 4 h and (D) IL-6 – 24 h.

**
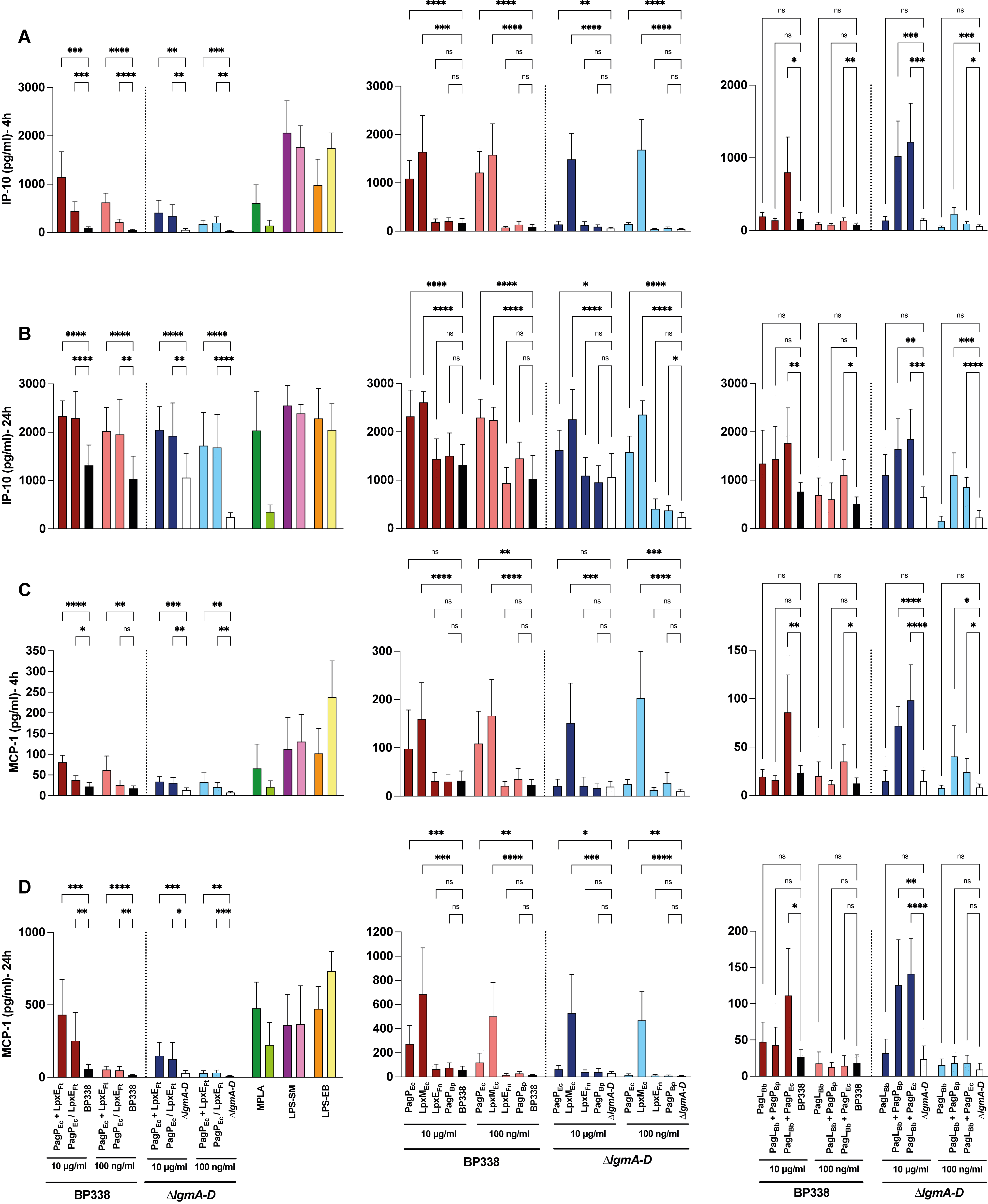
**

**Figure S4.** **Release of TRIF-dependent pathway cytokines.**

Secreted cytokines/chemokines upon stimulation of THP-1-derived macrophages with 2 different doses of LOS (10 𝜇𝑔/𝑚𝑙 and 100ng/ml) extracted from *B. pertussis* BP338, $\Delta$*lgmA-D* and the strains PagP_Ec_ + LpxE_Ft_, PagP_Ec_ / LpxE_Ft_, PagP_Ec_, LpxM_Ec_, LpxE_Ft_, PagP_Bp_, PagL_Bb_, PagL_Bb_ + PagP_Bp_, PagL_Bb_ + PagP_Ec_ in BP338 and $\Delta$*lgmA-D*, measured by ELISA at 4h and 24h. (A) IP-10 – 4 h, (B) IP-10 – 24 h, (C) MCP-1 – 4 h and (D) MCP-1 – 24 h.

**
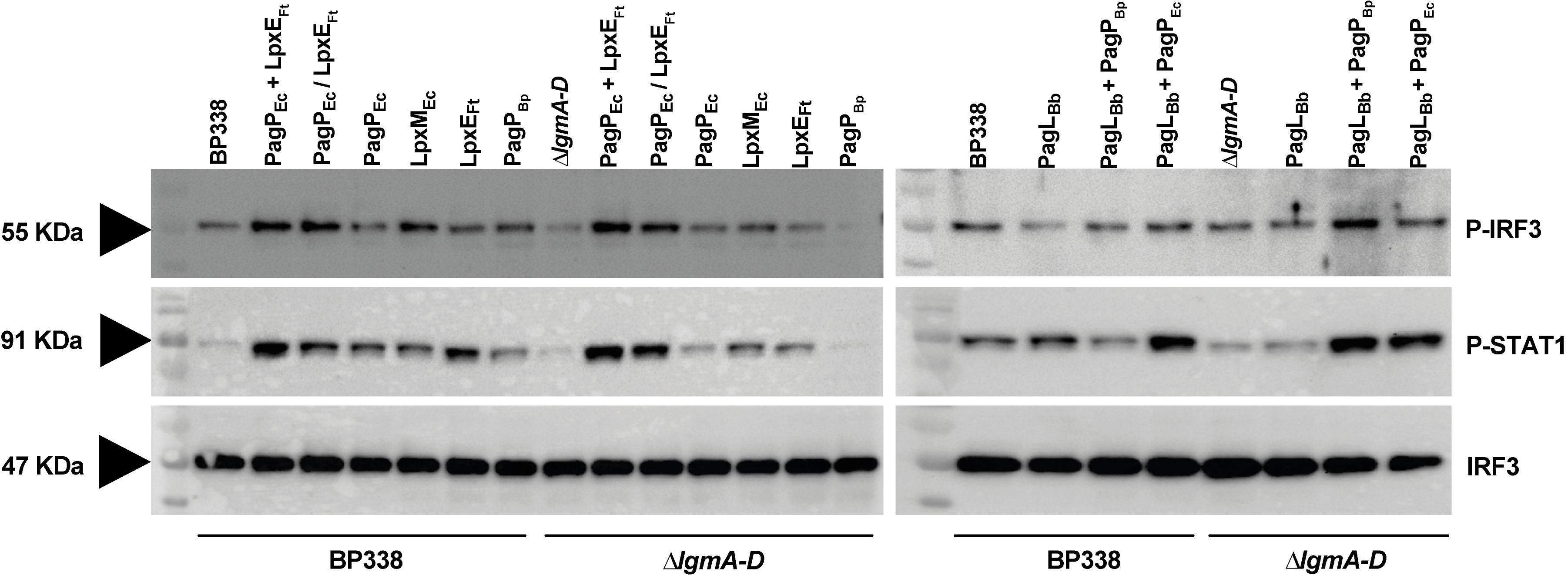
**

**Figure S5. Activation of TRIF pathway in human macrophages using heat-killed cells.**

THP-1-derived Macrophages were treated with heat-killed cells of the indicated constructs. The levels of phospho-IRF3, phospho-STAT1, and IRF3 were detected by immunoblotting. Results are from one of three representative experiments.

**
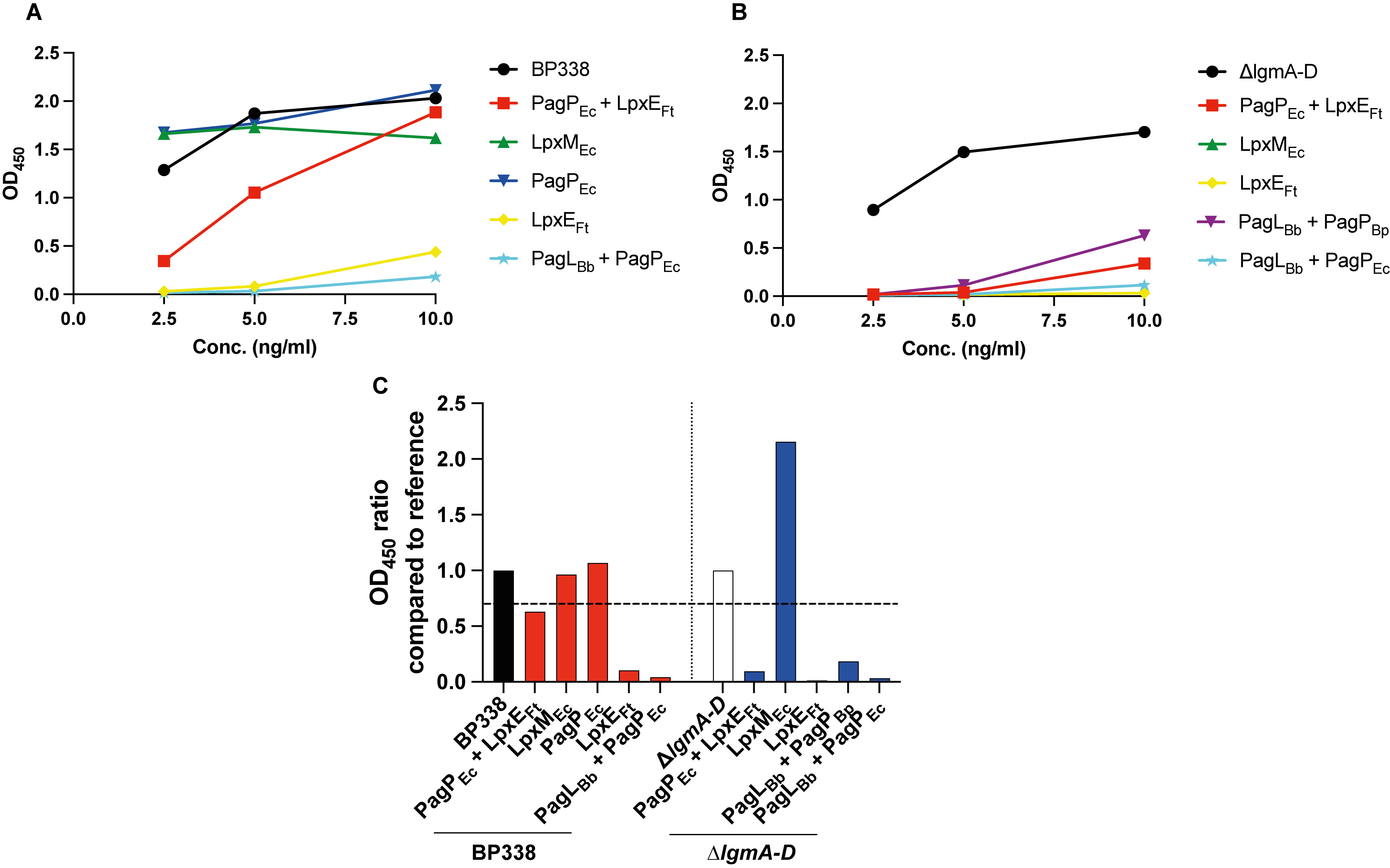
**

**Figure S6.** Release of IL-6 in terms of O.D values upon stimulation of PBMC with LOS extracted from the strains PagP_Ec_ + LpxE_Ft_, LpxM_Ec_, PagP_Ec_, LpxE_Ft_, and PagL_Bb_ + PagP_Ec_ in BP338 (A) and strains PagP_Ec_ + LpxE_Ft_, LpxM_Ec_, LpxE_Ft_, PagL_Bb_ + PagP_Bp_ and PagL_Bb_ + PagP_Ec_ in $\Delta$*lgmA-D* (B). An O.D ratio was calculated which represents the sum of the mean O.D of the 3 dilutions of the tested sample divided by the sum of the mean O.D of the 3 dilutions of the reference lot (C).

**
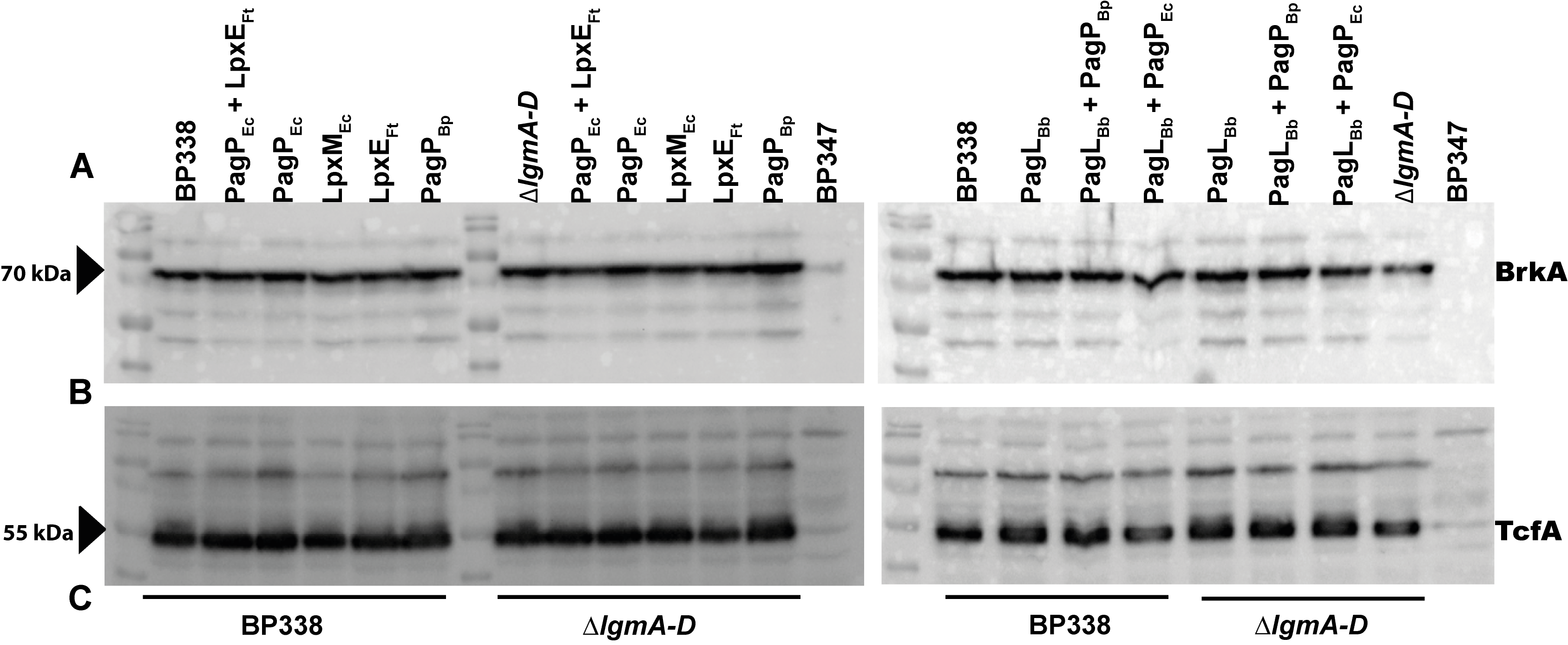
**

**Figure S7.** Western blot showing the expression of BrkA (A) and TcfA (B) and PageBlue stained gel showing the protein profile (C) from whole cell lysates of all *B. pertussis* BP338 strains with different lipid A variants constructed in this study in comparison to the Bvg- strain; BP347.

**Supplementary Methods:**

For BrkA and TcfA immunoblotting, we utilized an avirulent *B. pertussis* strain with an insertion in the *bvg* operon, as a negative control since it lacks positive regulation of BrkA and TcfA [45], due to the disrupted BvgAS locus [63]. Strains were grown in complete Stainer-Scholte medium to mid-log phase. One ml of culture was harvested by centrifugation and cell pellets were resuspended to an OD600 of 5.0 in PBS. Samples were mixed with SDS-PAGE sample buffer, heated to 100°C for 5 min, run on 12% SDS-PAGE and transferred to PVDF membranes. Membranes were probed with polyclonal antibodies raised against BrkA and TcfA [64, 65].
